# Bulk and Active Sediment Prokaryotic Communities in the Mariana and Mussau Trenches

**DOI:** 10.3389/fmicb.2020.01521

**Published:** 2020-07-14

**Authors:** Rulong Liu, Zixuan Wang, Li Wang, Zhenzhen Li, Jiasong Fang, Xing Wei, Wenxia Wei, Junwei Cao, Yuli Wei, Zhe Xie

**Affiliations:** ^1^Shanghai Engineering Research Center of Hadal Science and Technology, College of Marine Sciences, Shanghai Ocean University, Shanghai, China; ^2^State Key Laboratory of Geological Processes and Mineral Resources, Department of Earth Sciences, China University of Geosciences, Wuhan, China; ^3^Laboratory for Marine Biology and Biotechnology, Qingdao National Laboratory for Marine Science and Technology, Qingdao, China; ^4^Department of Natural Science, Hawaii Pacific University, Honolulu, HI, United States

**Keywords:** hadal trench, deepest ocean, novelty, activity, co-occurrence network, active community

## Abstract

Surprisingly high rates of microbial respiration have recently been reported in hadal trench sediment, yet the potentially active microorganisms and specific microbe–microbe relationships in trench sediment are largely unknown. We investigated the bulk and active prokaryotic communities and co-occurrence interactions of different lineages in vertically sectioned sediment cores taken from the deepest points of the Mariana and Mussau Trenches. Analysis on species novelty revealed for the first time the high rate of novel lineages in the microbial communities of the hadal trenches. Using 95, 97, and 99% similarity as thresholds, averagely 22.29, 32.3, and 64.1% of total OTUs retrieved from sediments of the two trenches were identified as the potentially novel lineages, respectively. The compositions of the potentially active communities, revealed via ribosomal RNA (rRNA), were significantly different from those of bulk communities (rDNA) in all samples from both trenches. The dominant taxa in bulk communities generally accounted for low proportions in the rRNA libraries, signifying that the abundance was not necessarily related to community functions in the hadal sediments. The potentially active communities showed high diversity and composed primarily of heterotrophic lineages, supporting their potential contributions in organic carbon consumption. Network analysis revealed high modularity and non-random co-occurrence of phylogenetically unrelated taxa, indicating highly specified micro-niches and close microbial interactions in the hadal sediments tested. Combined analysis of activity potentials and network keystone scores revealed significance of phyla Chloroflexi and Gemmatimonadetes, as well as several potentially alkane-degrading taxa in maintaining microbial interactions and functions of the trench communities. Overall, our results demonstrate that the hadal trenches harbor diverse, closely interacting, and active microorganisms, despite the extreme environmental conditions.

## Introduction

The deep ocean harbors around 75% of prokaryotic biomass and more than half of the prokaryotic production of the global ocean and is a key site for organic carbon remineralization and long-term carbon storage in the biosphere (Arístegui et al., 2009). Research efforts in the past decades have achieved dramatic advances on understandings of diverse deep-sea habitats, such as seamounts, cold seeps, brine pools, and hydrothermal vents (Corinaldesi, 2015). However, one type of deep-sea habitat, the hadal zone, is seldom touched due to the great technological challenges for investigation.

The hadal zone is the marine habitats with depth greater than 6,000 m. It is the deepest part of the ocean and is comprised primarily of deep trenches (Jamieson et al., 2010; Jamieson, 2015). This area is the end of the vertical transport of photosynthetic organic matter in the ocean and is also the “tunnel” for material and energy exchanges between the ocean and the deep earth, playing a special ecological role in the marine ecosystem (Jamieson, 2015; Liu et al., 2018b). The hadal zone is featured with many extreme physical–chemical conditions, such as low temperature and extremely high hydrostatic pressure (Taira et al., 2005; Jamieson et al., 2010). These environmental factors exert strong negative effects on cell growth and biological activities, causing great challenges to the survival of organisms (Mota et al., 2013; Tamburini et al., 2013). However, the hadal zone is not a “biological desert.” Multiple sources of organic matter inputs, combined with special topography and frequent tectonic activities, promote the accumulation of organic matter in the trenches (Ichino et al., 2015; Jamieson, 2015; Liu et al., 2018b). High content of organic matter, abundant microbial cells, and active microbial carbon turnover have been reported in the sediment of multiple trenches, making the hadal trenches “hot spots” of organic carbon degradation in the deep ocean (Danovaro et al., 2003; Glud et al., 2013; Wenzhöfer et al., 2016; Luo et al., 2018).

Despite the expanding reports about the active microbial carbon turnover in the trench sediments, limited information is known regarding the hadal microbial communities and the possible microbial processes that are driving the carbon cycles occurring in the hadal trenches. To date, only a handful studies have been conducted to investigate the diversity of microbial communities in water (Eloe et al., 2011; León-Zayas et al., 2015; Nunoura et al., 2015, 2016; Peoples et al., 2018; Liu et al., 2019) and sediment (Nunoura et al., 2018; Zhang et al., 2018; Cui et al., 2019; Peoples et al., 2019) of the hadal trenches. These studies revealed that the hadal zone contains a distinct composition of microbial communities, mainly enriched by heterotrophic taxa, such as members from phyla Marinimicrobia (SAR406 clade), Bacteroidetes, Chloroflexi (SAR202 clade), and Thaumarchaeota (Nunoura et al., 2015, 2016; Peoples et al., 2018; Liu et al., 2019). The findings seem to be well matched with the observed high rate of organic carbon turnover in the trenches (Nunoura et al., 2015; Tarn et al., 2016; Peoples et al., 2019). However, majority of the existing studies about hadal microbial communities utilized ribosomal RNA gene (rDNA) as their molecular target, which detect the bulk community including not only the metabolically active taxa but also the dormant, deceased taxa and even extracellular DNAs that persisted in the environments (Liu et al., 2015; Li et al., 2017). Recent studies have reported that only 6.5–34.5% of cells from pelagic microbial communities of the Mariana and Kermadec trenches were alive (Peoples et al., 2018). Whether the dominant taxa identified in the previous studies are alive and active in the hadal trenches is still an open question. In addition, it is still not known what kinds of microbe are alive and potentially the major contributor of microbial activities in the hadal zone and what the relative activities of different microbial taxa are in the alive community.

The composition and ecological functions of the microbial community are sustained by individual microorganisms via complex ecological interactions, such as cooperation, cross-feeding, competition, and predation (Faust and Raes, 2012). A better understanding of the biogeochemical cycling processes requires insights into the interactions among members within microbial communities (Faust and Raes, 2012; Reji et al., 2019). Reconstruction of an ecosystem-wide co-occurrence network has been applied to explore ecologically meaningful interactions between different microbial taxa in diverse environments (Williams et al., 2014; Cheung et al., 2018; Reji et al., 2019). To date, nothing is known regarding the interactions between different microbial taxa in the hadal trenches, and the relative significance of different taxa in maintaining the structure and functions of the hadal microbial community is not clear.

The trenches are typically V-shaped, comprising two slopes and the deeper trench floor, resulting in a distinct and elongated area in the seafloor, topographically separated from the upper ocean (Jamieson, 2011; Liu et al., 2018b). Such physical separation, combined with the extreme environmental conditions, was believed to result in many special and novel organisms in the hadal trenches (Jamieson et al., 2010; Jamieson, 2015). In supporting such hypothesis, a large number of new species have been reported for faunal communities in different trenches (Wolff, 1970; Jamieson, 2015). For example, as many as 250–310 new species have been found in 8 of the trenches explored during the Soviet *Vitjaz* and Danish *Galathea* expeditions, leading to the concept of “hadal community” (Wolff, 1959, 1970; Liu et al., 2018b) or “hadal biosphere” (Nunoura et al., 2015; Liu et al., 2018b). However, it has never been tested if a large number of novel lineages exist in the hadal microbial communities. In this study, we investigated for the first time the abundance, species composition, and spatial variations of the bulk and active prokaryotic community in sediment cores taken from the deepest points of the Mariana and Mussau trenches, using high-throughput sequencing on amplicons of both 16S rRNA gene and 16S rRNA, respectively. The objectives are to (1) determine the diversity and compositions of bulk and potentially active prokaryotic communities; (2) test the novelty of the hadal prokaryotic lineages; (3) compare the species composition and spatial variations of the potentially active prokaryotic communities in both trenches; and (4) study the relative activities and interactions of potentially active prokaryotic taxa in sediments of the hadal trenches.

## Materials and Methods

### Site Description and Sampling Procedure

Sediment samples were obtained from the Mariana and Mussau trenches during the cruise from December 2016 to January 2017 by the MV *Zhangjian*. The sampling site MT (11.4037°N, 142.3630°E), with water depth of 10,853 m, is close to the Challenger Deep of the Mariana Trench (Supplementary Figure S1). The site MST (0.8973°N, 148.8892°E), with water depth of 7,011 m, is located close to the deepest point of the Mussau Trench (Supplementary Figure S1). *In situ* O_2_ uptake rates in both sites were much higher than those from the abyssal plain, suggesting active microbial metabolism (Luo et al., 2018). Sediment samples were collected using a box corer (with a base area of 400 cm^2^ and a height of 25 cm) attached to the Hadal Lander II, developed recently by Shanghai Engineering Research Center of Hadal Science and Technology of Shanghai Ocean University (Luo et al., 2018). Two hours after the lander reached the seafloor, the sampling chamber was slowly driven into the seafloor until it reached around 21 cm below the sediment surface. A lid was then released to seal the box corer, and the lander was recovered. After recovering on board, the sediment samples were immediately subsampled using 50-mL sterile centrifuge tubes, and the tubes were stored under −80°C on board.

### DNA/RNA Extraction and Reverse Transcription

Sediment cores were thawed on ice and were depth fractioned to 0–2, 2–3, 3–4, 4–5, 5–6, 6–7, 7–8, 8–9 and 9–10 cm subsamples. Total DNA and RNA were co-extracted from triplicate 1-g sediments of each depth fraction using the PowerSoil^®^ Total RNA Isolation Kit and DNA Elution Accessory Kit (MoBio Lab, United States), with minor modifications: after the lysis step, synthetic 400-bp DNA and 383-bp RNA fragments, prepared according to Supplementary Material (see section “Supplementary Methodology”), were added to each sample (1 × 10^6^ copies each) as nucleic acid recovery control (NRC). The rest of the steps of the DNA/RNA extraction followed the manufacturer’s instructions, and the extracted nucleic acids were subsequently frozen at −80°C until use. Extraction blanks were included for each batch of extraction and did not result in detectable nucleic acids. The RNA samples were treated with DNase I and subjected to the synthesis of cDNA using the GoScript^TM^ Reverse Transcription System (Promega, United States) and random primers. The no-template and no-transcriptase control reactions included in each reverse transcription run did not result in detectable cDNA.

### Quantitative PCR (qPCR) Analyses

Each DNA and cDNA sample was tested with three SYBR^®^ Green qPCR assays to determine the copy numbers of bacterial and archaeal 16S rRNA and 16S rDNA, as well as copy numbers of NRCs. Details of primer sequences, *in silico* testing of specificities, and performance of each qPCR assay were provided in Supplementary Tables S1–S3. For procedures of the qPCR analysis, please refer to Supplementary Material section “Supplementary Methodology” and Liu et al. (2015). Percent recovery of the DNA and RNA NRCs was determined for each sample by dividing the measured copy numbers with total copies added (1 × 10^6^ copies). The copy numbers of the bacterial and archaeal 16S rDNA and 16S rRNA reported in the manuscript have been corrected by the percent recovery of DNA and RNA NRCs in each sample.

### PCR Amplification of 16S rRNA and rRNA Gene

DNA and cDNA samples were amplified with a barcoded primer set 515F/907R targeting V4–V5 hypervariable regions of the prokaryotic community (Supplementary Tables S1, S2). The detailed procedure of PCR reactions including reaction mixtures and thermocycle programs was the same as that of Liu et al. (2018a). Negative control and positive control were included in each amplification run. Within each depth fraction, triplicate PCR products were acquired for each molecular marker (DNA or cDNA), and they were then combined as two samples (DNA and cDNA). cDNAs from 6–7 to 8–9 cm of the MT site failed in PCR amplifications and were excluded from further analysis. Eight additional samples were prepared for DNA and cDNA from four depth fractions of MST, serving as replicates to monitor the variations between different runs of the PCR and the sequencing process. Ultimately, a total number of 42 samples (20 cDNA and 22 DNA samples) were successfully prepared and went through sequencing. PCR products from each sample were gel purified using AxyPrep DNA Gel Extraction Kit (Axygen Biosciences, Union City, CA, United States) and quantified using QuantiFluor^TM^-ST (Promega, United States).

### Illumina MiSeq Sequencing, Sequence Processing, and Diversity Analysis

Purified amplicons from different samples were equimolar pooled and subjected to paired-end sequencing (2 × 300) on an Illumina MiSeq platform (Illumina, San Diego, CA, United States) in Majorbio Bio-Pharm Technology Co. Ltd. (Shanghai, China). Raw fastq files were demultiplexed, quality-filtered by Trimmomatic (Bolger et al., 2014), and assembled by FLASH (Magoc and Salzberg, 2011). Chimeric sequences were identified and removed using UCHIME and operational taxonomic units (OTUs) were clustered with 97% similarity cutoff using UPARSE (version 7.1). The taxonomy of each OTU was assigned by RDP Classifier against the SILVA 16S rRNA database (SSU132) with a confidence threshold of 70%. Sequences found within our sequenced PCR negative control and those similar to known contaminants related with human sources or contaminants reported in previous hadal trench studies (Nunoura et al., 2015; Peoples et al., 2019) were discarded (please refer to Supplementary Material for full list). These processes resulted in 6,680–7,5476 sequences for different samples in the generated OTU table, and the sequences were resampled according to the minimum sequence number among all samples. Diversity indices including Sobs, Chao 1, Shannon index, Simpson index, and Good’s coverage were calculated using Mothur (version 1.35.1).

### Novelty of the Prokaryotic Taxa From the Hadal Trenches

The degree of novelty of the hadal prokaryotic lineages was assessed by comparing the detected 16S rRNA and rDNA sequences to those present in public databases. Representative sequences from each OTU were compared with the SILVA SSU Ref (release132) (Quast et al., 2013; Yilmaz et al., 2014) and NCBI Nucleotide (nt) databases (Wheeler et al., 2008), using 97 and 99% identity values as proxies of novel lineages. The 235-Mb SILVA SSU Ref and 72-G NCBI nt databases were downloaded on April 21, 2019, and served as reference for BLAST (v2.9.0). A cutoff *e*-value of 1E-05 was used, and 10 target sequences were allowed for each query sequence. The valid BLAST results were examined to find out novel OTUs that showed <95%, <97%, or <99% identities with existing sequences in public databases. The list of novel OTUs was extracted out, and their distribution and relative abundance in different MT or MST sediment prokaryotic communities were analyzed.

### Variations of Community Composition Between Different Trenches or Different Molecular Markers

Beta-diversity analysis was conducted to reveal variations of the prokaryotic communities in different depths, different trenches, and different molecular markers. To achieve a comprehensive understanding on hadal prokaryotic communities, published data of 38 rDNA libraries from sediment of Mariana Trench and Kermadec Trench were also included and were labeled as MT-ref. and KT-ref., respectively (Peoples et al., 2019). Raw sequence reads were downloaded from the NCBI SRA database and gone through the sequence processing procedure together with the raw sequences from this study. The generated OTU table was resampled to the minimum sequence number (5,667 sequences per sample). A Bray–Curtis similarity matrix was then generated based on microbial compositions at class level and was visualized using non-metric multidimensional scaling (nMDS) and hierarchical clustering (UPGMA) using PRIMER 6.0 package. One-way analysis of similarity (ANOSIM) was performed to test for differences between different trenches (groups MT, MST, MT-ref., and KT-ref.) or between different molecular targets (groups rDNA and rRNA). Significance levels of the differences between compared groups were tested using permutational methods with 1,000 permutations.

### Co-occurrence Network Analysis on Dominant Taxa From Both Trenches

Co-occurrence network analysis was conducted to investigate the interactions between potentially active prokaryotic lineages in sediment of the hadal trenches, based on the OTU table containing only the 20 rRNA libraries. To increase the sensitivity of the network, only OTUs that fulfilled the following three criteria were included in the network analysis: (1) with average relative abundances >0.05% in total sequence reads of all rRNA libraries, (2) present in >50% of total samples, and (3) present in at least 3 samples in each trench (Berry and Widder, 2014; Cheung et al., 2018). Correlations between OTUs were then calculated using the SparCC program with 20 iterations and 500 bootstraps (Friedman and Alm, 2012). Only the edges with significant (*p* < 0.001) and strong correlations (correlation coefficient > 0.500) were kept (Cheung et al., 2018), and the network was visualized by Cytoscape (version 3.7.1; Shannon et al., 2003). Topological parameters of the network were calculated using NetworkAnalyzer plugin (Assenov et al., 2008). The potential modules were identified with the Markov cluster (MCL) algorithm using clusterMaker2 plugin (Morris et al., 2011). Betweenness centrality and degree centrality scores were adopted as indexes to evaluate the significance of OTUs in the network, and the OTUs with the highest values of either betweenness centrality or degree centrality were identified as putative keystone taxa (Reji et al., 2019).

## Results

### Diversity and Composition of the Prokaryotic Communities in Hadal Sediments Revealed by 16S rDNA and rRNA

After sequence processing and resampling, our final data set contained 280,560 sequences in total (6,680 sequences/sample). The total numbers of OTUs retrieved were 1,456, 1,102, 1,833, and 1,927 from MT rDNA, MT rRNA, MST rDNA, and MST rRNA libraries, respectively. Analysis of alpha diversity suggests that both the species richness (e.g., Chao 1) and diversity indexes (Shannon) were higher in rDNA libraries than in rRNA libraries for the MT samples (Supplementary Figure S2 and Supplementary Table S4). In contrast, in MST samples, comparable Chao 1 values were observed for rDNA and rRNA libraries, whereas the Shannon index was obviously higher in rRNA libraries than rDNA libraries (Supplementary Figure S2 and Supplementary Table S4). The Good’s coverage ranged from 95.1 to 99.9% in all rDNA and rRNA libraries, with an average of 97.2% (Supplementary Table S4), suggesting that the majority of the diversity of the studied microbial communities was covered.

**FIGURE 1:**
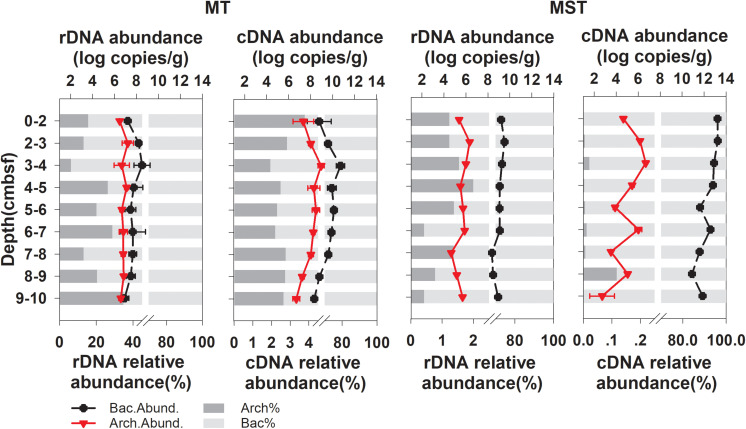
Changes in abundance (log transformed) and relative abundance of bacterial and archaeal rRNA (cDNA) and 16S rDNA at different depths of MT and MST sediments. Data points show the mean and standard deviations among triplicate samples of each depth fraction. Relative abundance of Archaea (Arch.%) and Bacteria (Bact.%) in total prokaryotic communities were calculated using mean abundance of Archaea or Bacteria in each depth fraction.

Quantitative PCR revealed that bacteria dominated the prokaryotic communities in all of the tested samples from the two trenches (Figure 1). The archaeal community only accounted for 6.12–34.72% and 0.42–1.99% of total prokaryotic rDNA copies and 1.94–3.78% and 0.00–0.12% of prokaryotic cDNA copies, in MT and MST sediments, respectively (Figure 1). The copy numbers of the prokaryotic rDNA and rRNA (cDNA) were the highest at the surface or near the surface of sediments and decreased rapidly with increasing depth (Figure 1).

**FIGURE 2:**
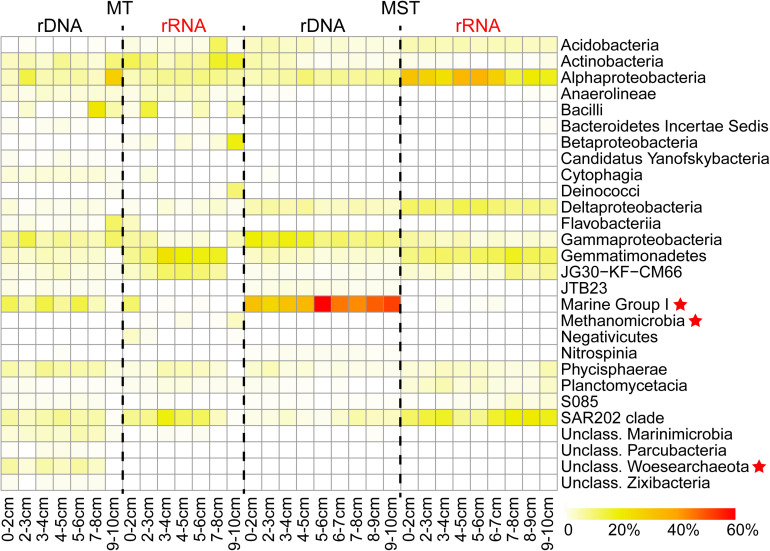
Composition of the total and potentially active prokaryotic communities in MT and MST sediments. The heatmap shows variations of the dominant classes in different samples. Only the classes which averagely account for >1% of the total sequence reads in rDNA or rRNA libraries from the same trench were shown. Archaeal classes were labeled with star symbols.

A sequence analysis of different rDNA and rRNA libraries showed that the prokaryotic communities from MT and MST sediment samples mainly consisted of taxa from 29 dominant classes in 16 phyla. Among them, 26 classes were bacteria and only 3 classes (unclassified Woesearchaeota, Methanomicrobia, and Marine Group I) were archaea (Figure 2). The rRNA libraries from MT sediment samples mainly consisted of classes Gemmatimonadetes, Actinobacteria, SAR202 clade, and Alphaproteobacteria and JG30-KF-CM66 clades and on average account for 51.53% of the total sequences. The rRNA libraries from MST sediments were dominated by classes of Alphaproteobacteria, SAR202 clade, Gemmatimonadetes, Deltaproteobacteria, and Acidobacteria, accounting for 70.97% of the total sequences from MST rRNA libraries (Figure 2). It is clear that the species composition of the rRNA libraries was drastically different from those of rDNA libraries from the same sediment sample in both trenches (Figure 2).

### Novel Prokaryotic Lineages in Hadal Sediments

**FIGURE 3:**
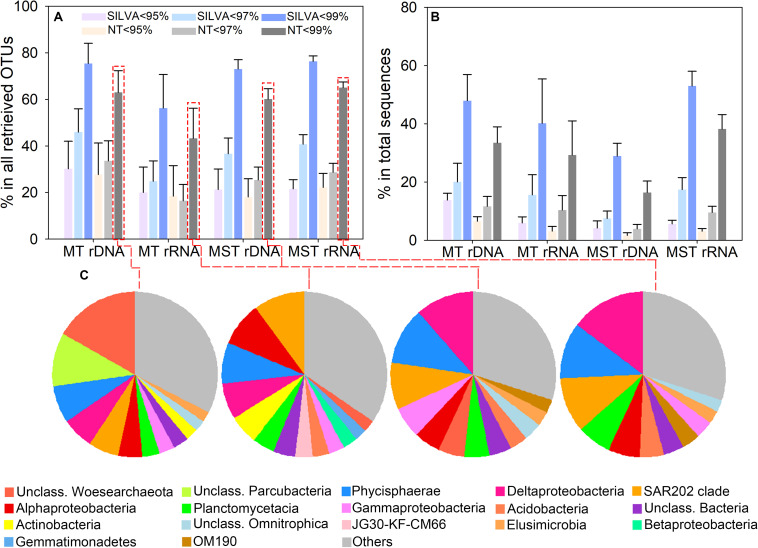
Identified novel OTUs based on BLAST analysis against the SILVA and NCBI nt databases. Novel lineages were defined using 95, 97, and 99% identity as thresholds, respectively. **(A)** Proportion of novel OTUs in the total number of OTUs retrieved from the rDNA and rRNA libraries of different trenches. **(B)** Relative abundance of the novel OTUs in the total sequence reads from rDNA and rRNA libraries of different trenches. The bar and error bar show the mean and the standard deviation among different samples from the same trench. **(C)** Taxonomy of the novel OTUs at class level, and the proportion of OTUs from each class in the total number of novel OTUs. OTUs that account for <2% of identified novel OTUs from rDNA and rRNA libraries of different trenches were classified as “Others.”

We assessed the degree of novelty of prokaryotic diversity in the hadal sediment by comparing the detected 16S rRNA gene sequences to those present in public databases. OTU representative sequences were compared with the SILVA and NCBI databases using 95, 97, and 99% identity values as thresholds. OTUs showed that identities <95%, <97%, or <99% with existing sequences in public databases were defined as potential novel OTUs. The result showed that averagely 22.29% (ranged from 18.11 ± 7.82% to 30.23 ± 11.77%) of OTUs from rDNA and rRNA libraries of both trenches were identified as novel OTUs at the 95% identity level (Figure 3A). Averagely 32.30% (ranged from 16.45 ± 7.00% to 45.97 ± 10.00%) of OTUs from rDNA and rRNA libraries were identified as novel OTUs at the 97% identity level (Figure 3A). When using 99% identity as threshold, novel OTUs averagely account for 64.08% (ranged from 43.27 ± 12.93% to 75.46 ± 8.63%) of total OTUs from different libraries (Figure 3A). The novel lineages defined at 95% identity averagely represent 5.01% (ranged from 1.65 ± 0.89% to 12.13 ± 4.16%) of total reads from rDNA and rRNA libraries of both trenches (Figure 3B). The sequences of novel lineages account for 3.91 ± 0.72% to 20.03 ± 6.42% (average value 12.11%) and 16.40 ± 2.67% to 53.01 ± 4.71% (average value 35.57%) of the total reads from different types of libraries from both trenches, at 97 and 99% identity thresholds, respectively (Figure 3B). These novel lineages belong to more than 30 phyla, within which Woesearchaeota, Parcubacteria, Planctomycetes, Chloroflexi, Proteobacteria, and Actinobacteria are the major groups (Figure 3C).

### Variations in Compositions of the Hadal Prokaryotic Communities Between Different Trenches or Different Molecular Markers

**FIGURE 4:**
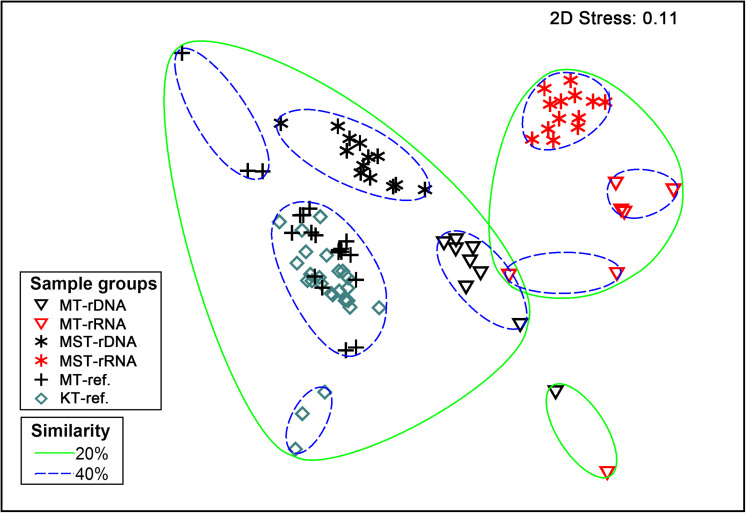
Non-parametric multidimensional scaling (nMDS) based on Bray–Curtis similarity of species composition at class level. Different symbols represent different sample types (rDNA vs. rRNA) from different trenches (MT, MST, MT-ref., and KT-ref.). Circles with solid and dashed lines indicate 20 and 40% similarity levels, respectively.

The nMDS based on all of the 42 rDNA and rRNA libraries in this study and 38 additional rDNA libraries from the literature (Peoples et al., 2019) revealed two large groups at the 20% similarity level: the rDNA group and the rRNA group (Figure 4). Within the two groups, several smaller groups were formed, separated by different trenches (Figure 4). ANOSIM analysis suggests significant differences between the observed groups (Table 1). It is interesting to note that the R values were significantly greater for comparisons between different molecular markers (rDNA vs. rRNA) than between different trenches using the same molecular targets (*P* < 0.050, *t*-test) (Table 1). In addition, when combining all rDNA samples from different trenches into one group, named as “All rDNA,” it showed a significant difference from the “All rRNA” group (ANOSIM, *R* = 0.864, *P* = 0.001, Table 1), suggesting again that the differences between the molecular markers were significantly larger than those between different trenches or between different depths.

**TABLE 1:** Analysis of similarity (ANOSIM) between different trenches (MT, KT, and MST) and different molecular targets (rDNA and rRNA), based on Bray–Curtis similarity of species composition at class level.

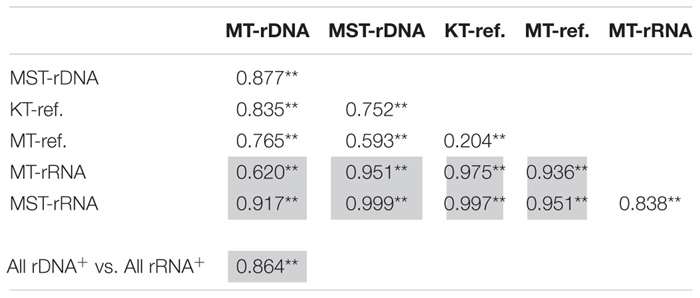

R-values were shown in the table to indicate the difference between each pair of comparison. The values with gray background are comparisons between different molecular targets, and those with white background are comparisons between different trenches using same molecular target. ** Significance levels P ≤ 0.001. ^+^All rDNA group includes MT-rDNA, MST-rDNA, MT-ref., and KT-ref. All rRNA group includes MT-rRNA and MST-rRNA.

**FIGURE 5:**
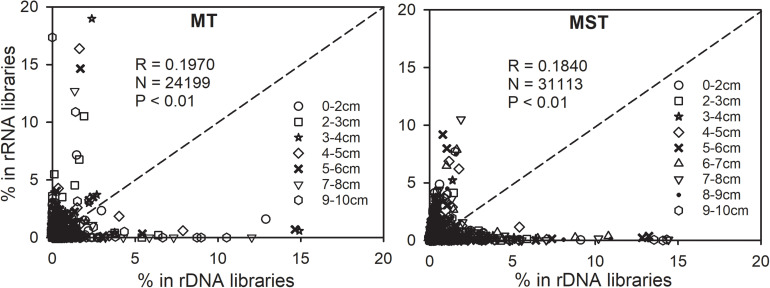
Relationships between 16S rRNA and rDNA frequencies of each OTU in rDNA and rRNA libraries of each individual sample in the MST and MT sediments. Different symbols show the depth of samples. The dotted line is the 1:1 line.

Relatedness between abundance and potential activities was further explored by plotting the relative abundance of OTUs in the rRNA library (rRNA frequency) against their relative abundance in the rDNA library (rDNA frequency) (Figure 5). Majority of the tested 24,199 and 31,113 data points are located off the 1:1 line. As a result, only weak correlations exist between rRNA and rDNA frequencies for OTUs from each sample tested in this study (Pearson correlation coefficient *R* = 0.1970 and 0.1840, Figure 5). It is important to notice that the most abundant prokaryotic lineages in the bulk communities of MT and MST sediments (rDNA libraries) generally showed low contribution to overall activities, as revealed by their low relative abundance in rRNA libraries (Figure 5 and Supplementary Figure S3). In contrast, the most active OTUs as indicated by their high percentage in rRNA libraries generally show low abundance in the bulk community (low percentage in rDNA libraries) (Figure 5).

### The Dominant Taxa in rRNA Libraries

**FIGURE 6:**
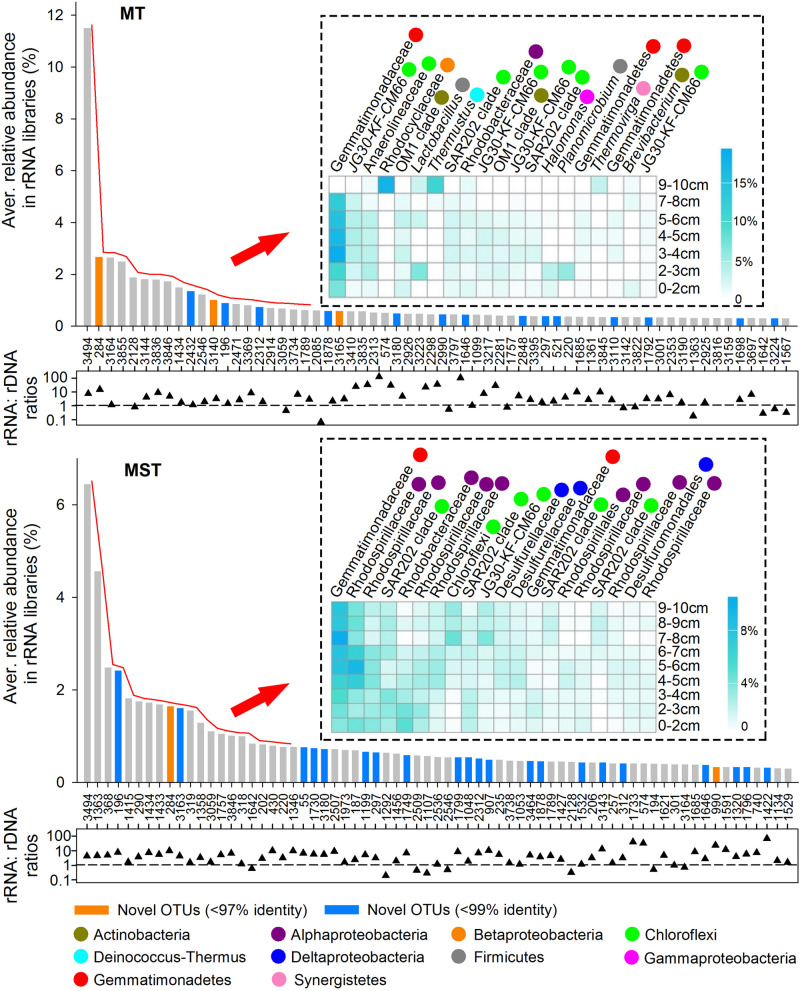
Dominant taxa in the potentially active prokaryotic communities in the sediments from MT and MST. The OTUs averagely representing ≥0.3% of total sequences among all rRNA libraries from the same trench were selected. The numbers below the *x*-axis are OTU names. Vertical bars show the average relative abundance of each OTU in the total sequences of all rRNA libraries from the same trench. The orange and blue bars indicate the novel OTUs identified at the 97 and 99% identity thresholds, respectively. Heatmaps show the top 20 most dominant taxa among all rRNA libraries of the same trench and their distributions at different depths. OTUs in the heatmap follow the same order with the *x*-axis of the bar chart. Labels beside the heatmap show the lowest identified taxonomy for each OTU. Colored circles show class/phylum that each OTU belongs to. The scattered triangles at the lower panel show log-transformed rRNA%: rDNA% ratio of each OTU, which was calculated using the average percentage of each OTU in rRNA libraries and rDNA libraries from the same trench. The dashed line indicates the 1:1 line.

In this study, dominant taxa were defined as OTUs that averagely contribute ≥0.3% of total sequences among all rDNA or rRNA libraries of the same trench. In total, 62 and 69 OTUs were identified as dominant taxa in rRNA libraries from MT and MST, respectively (Figure 6). These OTUs mainly belong to classes/phyla Gemmatimonadetes, Actinobacteria, SAR202 clade, JG30-KF-CM66, Alphaproteobacteria, Betaproteobacteria, Deltaproteobacteria, Gammaproteobacteria, Acidobacteria, Phycisphaerae, and Planctomycetacia, accounting for 54.73 and 60.47% of the total sequences in MT and MST rRNA libraries, respectively (Supplementary Figures S4, S5). The identified dominant taxa in rRNA libraries generally showed rRNA: rDNA ratios higher than 1, and some of the OTUs even showed rRNA: rDNA ratios higher than 100 (Figure 6).

The detailed classification and distribution of the top 20 most dominant OTUs in rRNA libraries of each trench were further analyzed (Figure 6). Generally, the most dominant OTUs in MT rRNA libraries were mainly uncultured OTUs belonging to Gemmatimonadaceae of phylum Gemmatimonadetes (3 OTUs), JG30-KF-CM66 (4 OTUs), Anaerolineaceae (1 OTU), and SAR202 clade (2 OTUs) of phylum Chloroflexi. The rest 10 OTUs were from phlya Proteobacteria, Actinobacteria, Firmicutes, Deinococcus–Thermus, and Synergistetes. The most dominant OTUs in MST rRNA libraries were less diverse than those in MT sediments. The OTUs were mainly from family Gemmatimonadaceae of phylum Gemmatimonadetes (2 OTUs); families Rhodospirillaceae (6 OTUs) and Rhodobacteraceae (1 OTU), and order Rhodospirillales (1 OTU) from Alphaproteobacteria; SAR202 clade (4 OTUs), JG30-KF-CM66 (1 OTU), and unclassified lineage (1 OTU) from phylum Chloroflexi; and family Desulfurellaceae (2 OTUs) and order Desulfuromonadales (1 OTU) from Deltaproteobacteria.

### Co-occurrence Patterns of Potentially Active Prokaryotic Taxa in Sediment of the Two Trenches

**FIGURE 7:**
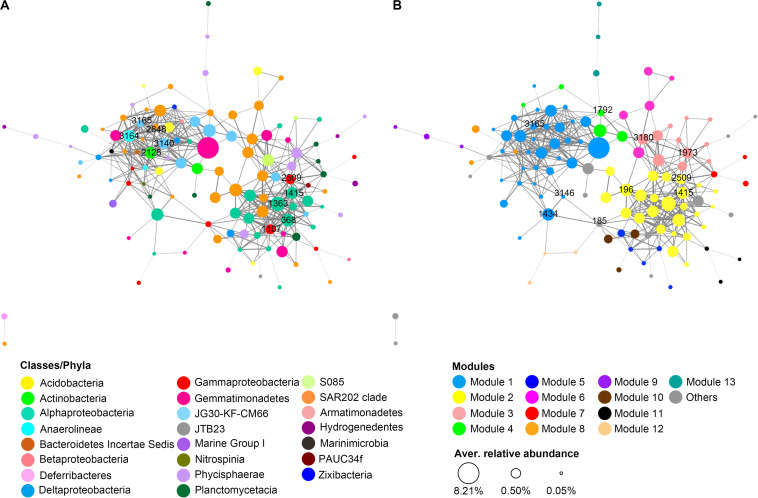
Co-occurrence network of the potentially active prokaryotic taxa in hadal sediments of MT and MST. Each node represents an OTU, and each edge represents a positive correlation. Nodes were colored according to **(A)** class level taxonomy or **(B)** modules predicted using the MCL algorithm. The size of the node indicates the average relative abundance of the OTU among all rRNA libraries, while the width of the edge is proportional to the level of correlation. Labeled numbers on nodes indicate the name of identified keystone OTUs. The keystone nodes with the highest degree centrality were labeled at network **(A)**, while those with the highest betweenness centrality were labeled at network **(B)**.

A total of 119 OTUs that were commonly present in rRNA libraries of both trenches went through the initial filtering step and were included in the network analysis. These OTUs are mainly from 15 phyla, among which Chloroflexi (40 OTUs), Proteobacteria (34 OTUs), Planctomycetes (17 OTUs), and Gemmatimonadetes (10 OTUs) are the largest group (Figure 7A). Markov cluster (MCL)-based modular analysis revealed that the network of potentially active prokaryotic communities from both trenches showed high modularity. There were 13 modules identified to contain ≥3 OTUs. Modules 1 and 2 are the largest clusters, containing around half of the OTUs in the network (Figure 7B). Generally, each module was comprised of OTUs from diverse prokaryotic lineages, and different modules have different compositions (Figures 7A,B). Among all taxa present in the network, OTUs from Chloroflexi (mainly SAR202 clade) are the most widely distributed, present in 8 of the 13 identified modules (Figure 7).

In the network, degree centrality and betweenness centrality scores were predicted as metrics of keystone taxa (Reji et al., 2019). The top 10 OTUs with the highest degree centrality scores were members of Anaerolineaceae (1 OTU), JG30-KF-CM66 (2 OTUs), SAR202 (1 OTU), Rhodospirillaceae (3 OTUs), Xanthomonadales (2 OTUs), and OM1 clade (1 OTU) (Figure 7A and Supplementary Table S5). OTUs with top 10 highest betweenness centrality scores belong to the SAR202 clade (4 OTUs), JG30-KF-CM66 (1 OTU), Rhodobacteraceae (1 OTU), Rhodospirillaceae (1 OTU), Xanthomonadales (1 OTU), BD7-8 marine group (1 OTU), and Phycisphaeraceae (1 OTU) (Figure 7A and Supplementary Table S5). Majority of the identified keystone OTUs showed high values in one type of centrality, i.e., degree centrality or betweenness centrality. However, OTU1414, OTU3165, and OTU2509 from uncultured Rhodospirillaceae, JG30-KF-CM66, and Xanthomonadales, respectively, showed high scores for both degree and betweenness centrality. All of the nodes with the highest degree centrality were located in the center of modules 1 and 2 (Figure 7A), while those with the highest betweenness centrality were widely distributed in different modules and usually located at the junctions of different modules (Figure 7B). Another observation is that the keystoneness of the OTUs was not related with their relative abundance. Majority of the identified keystone OTUs were not the most abundant OTUs in rRNA libraries.

## Discussion

### High Level of Novelty for Prokaryotic Communities in Hadal Sediments

This study assessed for the first time the novelty of prokaryotic communities in hadal sediments by comparing the detected 16S rRNA and rDNA sequences to those present in the SILVA and NCBI databases. Three identity values, i.e., 95, 97, and 99%, were selected as thresholds of potentially novel OTUs. The 95% identity is usually adopted as a proxy for genus, and 97% identity is widely used in microbial ecology studies as a broad proxy for identifying new species (Salazar et al., 2016). However, it is well known that 16S rRNA genes from different species may also show identities higher than such value (Fox et al., 1992; Acinas et al., 2004; Stackebrandt, 2006). New thresholds of 98.2–99.0% have been proposed and widely applied for identification of potentially new species (Stackebrandt and Ebers, 2006; Meier-Kolthoff et al., 2013; Kim et al., 2014). Therefore, the 97% identity value was used in this study as a conservative threshold to identify the new species, and the 99% identity value was utilized to identify all putatively new lineages/OTUs (Salazar et al., 2016).

Our results showed that averagely 22.29, 32.30, and 64.08% of OTUs from the rDNA and rRNA libraries of both trenches were identified as novel OTUs at 95, 97, and 99% identity levels, respectively (Figure 3A). The proportion of novel lineages in prokaryotic communities of the hadal sediment was comparable to those of the global bathypelagic microbial communities (Salazar et al., 2016). However, the novel lineages detected in this study averagely account for 5.01, 12.11, and 35.57% of total reads at 95, 97, and 99% identity thresholds, respectively (Figure 3B). Such finding is different from other marine habitats, where the novel lineages usually represent minor fractions of the total reads and have been considered as members of the “rare biosphere” (Sogin et al., 2006; Salazar et al., 2016; Mo et al., 2018). For example, the novel lineages only represented 2.2, 4.5, and 9.1% of total sequences in the global bathypelagic oceans, at 95, 97, and 99% identities, respectively (Salazar et al., 2016), and represent 5.5% of total sequences in global epipelagic and mesopelagic waters from the Tara Ocean samples (at 97% identity threshold; Sunagawa et al., 2015). The relatively high abundance of novel lineages in the trench sediment suggests their potential significance in activity and functions of hadal microbial communities and also demonstrates that the hadal trenches may be a reservoir of new microbial species. These novel lineages were with high diversity and belong to more than 30 phyla, including many understudied phyla such as Parcubacteria, Planctomycetes, Chloroflexi, and Woesearchaeota (Figure 3C). Further exploration on phylogeny and genomic features of these novel hadal lineages will greatly expand our current understanding on the diversity, ecological function, and life strategies of trench microorganisms.

### Significant Difference Between rDNA and rRNA Revealed Diversities

Compared with rDNA, ribosomal RNA (rRNA) is generally more related with activity states of the cells, as rRNA molecules are degraded rapidly in physically damaged or dead cells (Blazewicz et al., 2013; Maiväli et al., 2013; Liu et al., 2015) and its abundance is positively correlated with cellular activities such as metabolism and growth (Campbell et al., 2011; Hunt et al., 2013). On the other hand, dormant cells of certain microbial taxa were also reported to contain measurable amounts of rRNA in some cases (Blazewicz et al., 2013). Therefore, quantification and sequencing of rRNA detect the “potentially active” fractions of the microbial community. The ratios between rRNA and rDNA sequences (rRNA: rDNA ratio) can be further applied as a means of normalized rRNA transcripts against cell count to identify the metabolically active taxa (Steven et al., 2017). rRNA sequences and rRNA: rDNA ratios have been widely applied to characterize the active communities in different marine habitats, such as salt mash sediment (Kearns et al., 2016), surface seawater (Campbell et al., 2011), deep-sea water (La Cono et al., 2009; La Cono et al., 2015), deep-sea sediment (Yanagawa et al., 2013; Hoffmann et al., 2017), and ice sheet (Stibal et al., 2015).

Our results revealed significant differences between species composition of the rDNA and rRNA libraries from sediments of hadal trenches, and the differences between molecular markers (i.e., rDNA vs. rRNA) were even larger than those between different depths (0–10 cm), different trenches (MT, MST, and KT-ref.), or different sampling times (MT and MT ref.) (Figure 4 and Table 1). Significant differences between the rDNA and rRNA revealed that diversities have also been widely reported in microbial communities from other environments (La Cono et al., 2009, 2015; Campbell et al., 2011; Yanagawa et al., 2013; Stibal et al., 2015; Kearns et al., 2016; Hoffmann et al., 2017). However, the rRNA and rDNA relationships of the microbial communities from hadal trenches showed features that are drastically different from those of other environments. For example, strong correlations were observed between RNA and DNA frequencies among different taxa in surface seawater, marine sediments, or Arctic thermokarst lakes, although overall species compositions from rRNA and rDNA libraries were significantly different (Campbell et al., 2011; Matheus Carnevali et al., 2018). In contrast, for hadal microbial communities tested in this study, correlations between rRNA and rDNA frequencies of different taxa were very weak (*R* < 0.2000, Figure 5), suggesting that the potential activity of an OTU in hadal sediment does not follow its relative abundance in the bulk community. Peoples et al. (2018) reported that only 6.5–34.5% of microbial cells from hadal waters were active, suggesting that majority of the cells in hadal microbial communities are either dead or with activities lower than the detection limit of the method utilized (biorthogonal non-canonical amino acid tagging). The existence of a large number of low-activity/dormant or dead cells in the hadal habitats could be a possible reason for weak correlations between rRNA and rDNA frequencies we observed in the hadal sediments.

Importantly, many of the most dominant taxa in the bulk communities analyzed in this study showed an unproportionally low contribution to community activities as revealed by their low percentage in corresponding rRNA libraries (Figures 3, 5 and Supplementary Figure S3). These taxa mainly belong to *Candidatus Nitrosopumilus* and Marine Group I of Thaumarchaeota, SAR406 clade of Marinimicrobia, and Flammeovirgaceae of Bacteroidetes (Supplementary Figure S3), which have also been reported to be the dominant taxa in rDNA libraries from hadal sediments in previous studies (Nunoura et al., 2018; Cui et al., 2019; Peoples et al., 2019). Therefore, caution must be exercised when interpreting the possible contributions of these abundant taxa to the overall ecological functions of the hadal prokaryotic communities. Indeed, Peoples et al. (2018) found that the isolates of several dominant bacterial taxa in rDNA libraries of hadal trenches cannot grow at pressures greater than 40 MPa, but they remained intact for long terms under extremely high pressure (90 MPa), suggesting that these bacteria may exist in the hadal trenches but with low activity or in dormant state. Currently, hadal microbial communities have been mainly studied via DNA-based analysis, which may overestimate the importance of some numerically abundant but less active taxa. We propose that the combination of the rRNA-based diversity analysis and DNA-based technologies such as metagenomic analysis would greatly improve the understanding on metabolic activity and function of the hadal microbial communities.

A concern regarding RNA-based technologies is the possible degradation of RNA during sample recovery from deep sea, due to changes in environmental conditions such as temperature and pressure. However, recovery of deep-sea water (∼3,000 m depth) and onboard processing within 24 h have been shown to cause only slight changes in rRNA diversity, by comparing with pressure-retained samples or samples filtered *in situ* (La Cono et al., 2009, 2015). In this study, sample recovery via landers and onboard sample processing (i.e., sediment coring and subsampling) usually took <5 h; the rRNA diversities are therefore expected to have limited changes from their *in situ* conditions. Nevertheless, the possible impacts due to the potentially larger extent of environmental changes for sample recovery from hadal depth than those from 3,000 m need to be further addressed (Wang et al., 2019).

### The Active Prokaryotic Taxa in the Sediment of Hadal Trenches

High rates of microbial carbon turnover have been widely reported in the bottom sediment of multiple trenches (Glud et al., 2013; Wenzhöfer et al., 2016; Luo et al., 2018). Our results showed that the potentially active prokaryotic communities which possibly contribute to the observed biogeochemical activities in the hadal sediments were highly diversified, as revealed by the high level of species richness and diversity of rRNA libraries (Supplementary Figure S2 and Supplementary Table S4). To discover the major prokaryotic taxa that potentially contribute to the observed biogeochemical activities in hadal trenches, dominant OTUs in rRNA libraries of the both trenches were identified (Figure 6). These OTUs generally showed rRNA: rDNA ratios greater than 1, and even higher than 100 for some of the OTUs (Figure 6). According to the models of Steven et al. (2017), these OTUs represent metabolically active taxa of prokaryotic communities in the tested hadal sediment of the two trenches. The dominant OTUs identified in rRNA libraries mainly belong to classes/phyla Gemmatimonadetes, Actinobacteria, SAR202 clade, JG30-KF-CM66, Alphaproteobacteria, Betaproteobacteria, Deltaproteobacteria, Gammaproteobacteria, Acidobacteria, Phycisphaerae, and Planctomycetacia (Figure 6 and Supplementary Figures S4, S5). Members of these taxa have been reported to perform aerobic or anaerobic degradation of diverse types of organic matter, including proteins, fatty acids, cellulose, chitin, pectin, fluoranthene, phenanthrene, and many aromatic compounds (Dai et al., 2005; Oren, 2014; Liang et al., 2015; Smalley et al., 2015; Chen et al., 2016; Landry et al., 2017), supporting their potential contributions to the observed high rates of microbial carbon turnover in hadal sediments (Glud et al., 2013; Luo et al., 2018).

### Co-occurrence of Potentially Active Prokaryotic Taxa in the Hadal Sediments

To our knowledge, this is the first study exploring the co-occurrence network and modular patterns of potentially active sediment prokaryotic communities in hadal trenches. Markov cluster (MCL)-based modular analysis revealed as many as 13 modules that contain ≥3 OTUs (Figure 7B), suggesting that highly diversified micro-niches and structured microbial communities exist in sediment of the hadal trenches. The topology of the network exhibited a high level of modularity, similar to those constructed from other complex environments, such as soil (Williams et al., 2014), wetland sediment (Cheung et al., 2018), and seawaters (Reji et al., 2019). Each module was comprised of OTUs from multiple unrelated prokaryotic taxa that are not necessarily associated at phylogenetic closeness (Figures 7A,B). For example, the largest module 1 was mainly comprised of phyla Chloroflexi, Acidobacteria, Gemmatimonadetes, and Proteobacteria, with 1 or 2 OTUs from each of the additional phyla Armatimonadetes, Bacteroidetes, Marinimicrobia, Nitrospinae (Nitrospinaceae), Planctomycetes, Zixibacteria, and Thaumarchaeota (Nitrosopumilus) (Figure 7). Co-occurrence of the different microbial taxa in the same module suggests that they may have similar nutritional preferences or similar eco-physiological responses to varying environmental conditions at different depths of the sediment or have close metabolic interactions with each other (Faust and Raes, 2012; Reji et al., 2019).

There were varied criteria to identify the keystone taxa in the co-occurrence network, including solely based on highest betweenness centrality (Banerjee et al., 2016) or based on combined scores of high degree centrality, high closeness centrality, and low betweenness centrality (Berry and Widder, 2014). In this study, keystone taxa in the modules were identified based on both degree centrality and betweenness centrality scores, following recent studies in oceanic habitats (Reji et al., 2019). The taxa with high degree centrality values are “hub” nodes that have many connections in the network, while those with high betweenness centrality values serve as “bridges” between subnetworks or modules and are important for the flow of information between different parts of the network (Reji et al., 2019). The “hubs” and “bridges” identified in this study mainly belong to classes Anaerolineaceae, JG30-KF-CM66, SAR202, Alphaproteobacteria (Rhodobacteraceae and Rhodospirillaceae), Gammaproteobacteria (Xanthomonadales), Phycisphaerae, and Actinobacteria (OM1 clade) (Figure 7 and Supplementary Table S5). Although many of those keystone OTUs are not the most abundant taxa in the rRNA libraries, they occupy the important localities in the network and therefore may play key roles in mediating network interactions and maintaining the overall metabolic functions of the active microbial communities in sediments of the hadal trenches tested (Banerjee et al., 2016; Cheung et al., 2018).

### Phyla Chloroflexi and Gemmatimonadetes Are Important in Mediating the Activity and Functions of Trench Prokaryotic Communities

Around half of the dominant OTUs in rRNA libraries of both trenches belong to Gemmatimonadetes (class Gemmatimonadetes) and Chloroflexi (classes SAR202, Anaerolineae, and JG30-KF-CM66) (Figure 6 and Supplementary Figures S4, S5), making them the top phyla of the potentially active communities in the hadal sediments tested (Figure 2). Gemmatimonadetes and Chloroflexi have been repeatedly reported to be dominant groups in rDNA libraries from sediment of the Mariana Trench and other trenches (Nunoura et al., 2018; Cui et al., 2019; Peoples et al., 2019). Our results demonstrate that these taxa not only are numerically abundant but also have high activity potentials in the prokaryotic communities of MT and MST sediments. These results suggest the important role of Gemmatimonadetes and Chloroflexi in driving the organic carbon turnover in the hadal trenches. Both phyla encompass a tremendously wide range of metabolic strategies from photosynthesis to aerobic or anaerobic heterotrophy (DeBruyn et al., 2011; Zeng et al., 2014; Landry et al., 2017; Mehrshad et al., 2018a, b). The metabolic versatility of these taxa might be a possible reason for their significance in hadal trenches where organic matters are from complex sources and with high level of recalcitrance (Jamieson, 2015; Liu et al., 2018b).

In addition to high abundance and activity potentials, Chloroflexi members were also important in mediating the interaction among different prokaryotic taxa in the tested hadal trenches, as around half of the keystone OTUs identified in the co-occurrence network belong to this phylum (Figure 7 and Supplementary Table S5). Particularly, OTUs from the SAR202 clade were found to be widely distributed in 8 of the 13 identified modules in the network (Figures 7A,B), suggesting great niche separations among different SAR202 lineages. On the other hand, however, the majority of these SAR202 OTUs were connected with each other (Figure 7A), and 4 of the 10 OTUs with the highest betweenness centrality values belong to the SAR202 clade, serving as important “bridges” in connecting different modules. For example, OTU3180 connected with as many as 4 modules (modules 2, 3, 4, and 6). OTU1792 was the only node connecting with a remote module (module 13) in the major body of the network (modules 1, 3, and 4). The synchronous occurrence of wide distribution, close intra-clade connection, and high betweenness centrality were not observed for other classes in the network, making the SAR202 clade a key group of bacteria potentially mediating interactions between different niches in the surface sediment of the hadal trenches tested in this study. In fact, SAR202 have been reported to have diverse metabolic pathways that may link the biogeochemical cycles of carbon, nitrogen, and sulfur (Thrash et al., 2017; Colatriano et al., 2018; Mehrshad et al., 2018a). SAR202 lineages were also reported to develop specialized metabolism to oxidize different groups of recalcitrant organic compounds, via expansion of different combinations of paralogous genes (Landry et al., 2017; Saw et al., 2019). The diversified metabolisms and capability to degrade various groups of recalcitrant organic matter would allow SAR202 to adapt different niches, linking different biogeochemical reactions, and could be a possible reason for the important role of SAR202 clade in maintaining network structure and mediating interactions in prokaryotic communities in the sediment of hadal trenches.

### Alkane Degradation as a Potentially Important Pathway in Carbon Turnover of Hadal Trenches

A recent study by Liu et al. (2019) reported that the pelagic microbial communities in bottom seawaters of the Mariana Trench were enriched with alkane-degrading bacteria (*Oleibacter*, *Thalassolituus*, and *Alcanivorax*) and alkane-degrading activities. However, the alkane-degrading taxa identified in Liu et al. (2019) were not detected in sediment rDNA or rRNA libraries of this study. Instead, we identified several OTUs in sediments of the Mariana Trench, belonging to *Planomicrobium*, Xanthomonadales, Anaerolineaceae, and SAR202 clade (Figure 6 and Supplementary Table S5), members of which have been reported to be active or as potential alkane-degraders in aerobic or anaerobic conditions (Engelhardt et al., 2001; Head et al., 2006; Liang et al., 2015; Gutierrez, 2017; Landry et al., 2017). OTUs from *Planomicrobium*, Anaerolineaceae, and SAR202 were among the top 20 most dominant members in the rRNA libraries of MT samples (Figure 6), and members of Anaerolineaceae and Xanthomonadales were identified as keystone taxa in the co-occurrence network of potentially active prokaryotic community (Figure 7A and Supplementary Table S5), suggesting the important role of these hydrocarbon-degrading bacteria in supporting the function and structure of active prokaryotic communities in the sediment of MT. On the other hand, abundant n-alkane, potentially sourced from bacteria, algae, or contamination of diesel fuels, has been recently reported in surface sediments of the Mariana Trench (Guan et al., 2019; Liu et al., 2019), and the concentrations of alkanes increased along the down slope of the trench (Guan et al., 2019). The high activity potentials and keystone roles of the potential hydrocarbon-degrading bacteria, together with the abundant alkane content in sediment, led us to postulate that alkane degradation might be an important pathway of carbon turnover in the sediment of the Mariana Trench and are likely mediated by different taxa in the sediment than in the water column of the trench.

## Conclusion

With the expanding discovery of high content of sedimentary organic matter and active microbial carbon turnover from trenches, the significance of hadal microbes in deep-sea carbon cycle, as well as their contributions to phylogenetic and functional diversities of the marine prokaryotic communities, cannot be ignored. This study examined for the first time the “potentially active” sediment prokaryotic communities in hadal trenches of the western Pacific and demonstrated the high proportions of novel lineages existing in trench microbial communities, supporting the hypothesis that topographical isolation and extreme environmental conditions lead to new species in the hadal zone (Jamieson et al., 2010; Jamieson, 2015). The potentially active prokaryotic communities exhibited significantly different compositions from the bulk communities. In addition, many numerically abundant taxa in the bulk communities were found to have unproportionally low activity potentials, suggesting the decoupling between the abundance of microbial taxa and their ecological functions in the hadal environment. Our results also revealed the importance of members from classes Gemmatimonadetes, Actinobacteria, SAR202 clade, JG30-KF-CM66, Alphaproteobacteria, Gammaproteobacteria, and Phycisphaerae in maintaining metabolic activity and structural stability of the prokaryotic communities in sediment of the hadal trenches, for their dominance in rRNA libraries, high rRNA: rDNA ratios, or high keystone scores in the network interactions. Many of these classes, such as Gemmatimonadetes, SAR202, and JG30-KF-CM66, are largely understudied, despite their high abundance and prevalence in the marine habitats (Zeng et al., 2014; Mehrshad et al., 2018a, b). Further studies should be direct to explore the metabolic pathways, *in situ* activities, and adaptation strategies of these important taxa in the extreme environmental conditions of the hadal zone. Finally, this study is a snapshot of the microbial processes in sediment of the two trenches. Both the rRNA content and rRNA: rDNA ratios of different taxa observed in our results may be subject to changes at temporal or spatial scale, due to the variable supply of organic matter and their heterogenic distribution in the hadal trenches (Jamieson, 2015; Stewart and Jamieson, 2018), as well as different growth strategies of microbial taxa (R vs. K strategies) in response to perturbations of nutrient conditions (Roller et al., 2016). More studies with larger spatial and temporal scales, combined with hypothesis-driven cultivation experiments, are needed to further explore the biogeography, relative activities, and potential controlling factors for different microbial taxa that are metabolically active in the hadal trenches.

## Data Availability Statement

The datasets generated for this study can be found in the NCBI Sequence Read Archive accession ID SRP214992.

## Author Contributions

RL, LW, and JF designed the expedition and sampling scheme. ZW, ZL, XW, and WW conducted experimental procedures including DNA and RNA co-extraction, reverse transcription, PCR amplification, and qPCR. RL, ZW, and LW performed bioinformatical analyses including sequence processing, Blast, diversity analysis, and co-occurrence network analysis. RL, ZW, and JF analyzed and summarized the data and wrote the article. JC, YW, and ZX made comments and suggestions to the text. All authors contributed to the article and approved the submitted version.

## Conflict of Interest

The authors declare that the research was conducted in the absence of any commercial or financial relationships that could be construed as a potential conflict of interest.

## References

[B1] Acinas S. G. Klepac-Ceraj V. Hunt D. E. Pharino C. Ceraj I. Distel D. L. (2004). Fine-scale phylogenetic architecture of a complex bacterial community. *Nature* 430 551–554. 10.1038/nature02649 15282603

[B2] Arístegui J. Gasol J. M. Duarte C. M. Herndld G. J. (2009). Microbial oceanography of the dark ocean’s pelagic realm. *Limnol. Oceanogr.* 54 1501–1529. 10.4319/lo.2009.54.5.1501

[B3] Assenov Y. Ramirez F. Schelhorn S. E. Lengauer T. Albrecht M. (2008). Computing topological parameters of biological networks. *Bioinformatics* 24 282–284. 10.1093/bioinformatics/btm554 18006545

[B4] Banerjee S. Baah-Acheamfour M. Carlyle C. N. Bissett A. Richardson A. E. Siddique T. (2016). Determinants of bacterial communities in Canadian agroforestry systems. *Environ. Microbiol.* 18 1805–1816. 10.1111/1462-2920.12986 26184386

[B5] Berry D. Widder S. (2014). Deciphering microbial interactions and detecting keystone species with co-occurrence networks. *Front. Microbiol.* 5:219 10.3389/fmicb.2014.00219 PMC4033041 24904535

[B6] Blazewicz S. J. Barnard R. L. Daly R. A. Firestone M. K. (2013). Evaluating rRNA as an indicator of microbial activity in environmental communities: limitations and uses. *ISME J.* 7 2061–2068. 10.1038/ismej.2013.102 23823491 PMC3806256

[B7] Bolger A. M. Lohse M. Usadel B. (2014). Trimmomatic: a flexible trimmer for Illumina sequence data. *Bioinformatics* 30 2114–2120. 10.1093/bioinformatics/btu170 24695404 PMC4103590

[B8] Campbell B. J. Yu L. Heidelberg J. F. Kirchman D. L. (2011). Activity of abundant and rare bacteria in a coastal ocean. *Proc. Natl. Acad. Sci. U.S.A.* 108 12776–12781. 10.1073/pnas.1101405108 21768380 PMC3150899

[B9] Chen P. Zhang L. Guo X. Dai X. Liu L. Xi L. (2016). Diversity, Biogeography, and Biodegradation Potential of Actinobacteria in the Deep-Sea Sediments along the Southwest Indian Ridge. *Front. Microbiol.* 7:1340 10.3389/fmicb.2016.01340 PMC5002886 27621725

[B10] Cheung M. K. Wong C. K. Chu K. H. Kwan H. S. (2018). Community Structure, Dynamics and Interactions of Bacteria, Archaea and Fungi in Subtropical Coastal Wetland Sediments. *Sci. Rep.* 8:14397 10.1038/s41598-018-32529-5 PMC6158284 30258074

[B11] Colatriano D. Tran P. Q. Guéguen C. Williams W. J. Lovejoy C. Walsh D. A. (2018). Genomic evidence for the degradation of terrestrial organic matter by pelagic Arctic Ocean Chloroflexi bacteria. *Commun. Biol.* 1:90 10.1038/s42003-018-0086-7 PMC6123686 30271971

[B12] Corinaldesi C. (2015). New perspectives in benthic deep-sea microbial ecology. *Front. Mar. Sci.* 2:17 10.3389/fmars.2015.00017

[B13] Cui G. Li J. Gao Z. M. Wang Y. (2019). Spatial variations of microbial communities in abyssal and hadal sediments across the Challenger Deep. *PeerJ* 7:e6961 10.7717/peerj.6961 PMC6526897 31149407

[B14] Dai X. Wang Y. N. Wang B. J. Liu S. J. Zhou Y. G. (2005). *Planomicrobium chinense* sp. nov., isolated from coastal sediment, and transfer of *Planococcus psychrophilus* and *Planococcus alkanoclasticus* to *Planomicrobium* as *Planomicrobium psychrophilum* comb. nov. and *Planomicrobium alkanoclasticum* comb. nov. *Int. J. Syst. Evol. Microbiol.* 55 699–702. 10.1099/ijs.0.63340-0 15774646

[B15] Danovaro R. Croce N. D. Dell’Anno A. Pusceddu A. (2003). A depocenter of organic matter at 7800-m depth in the SE Pacific Ocean. *Deep Sea Res. Part I Oceanogr. Res. Pap.* 50 1411–1420. 10.1016/j.dsr.2003.07.001

[B16] DeBruyn J. M. Nixon L. T. Fawaz M. N. Johnson A. M. Radosevich M. (2011). Global biogeography and quantitative seasonal dynamics of Gemmatimonadetes in soil. *Appl. Environ. Microbiol.* 77 6295–6300. 10.1128/AEM.05005-11 21764958 PMC3165389

[B17] Eloe E. A. Shulse C. N. Fadrosh D. W. Williamson S. J. Allen E. E. Bartlett D. H. (2011). Compositional differences in particle-associated and free-living microbial assemblages from an extreme deep-ocean environment. *Environ. Microbiol. Rep.* 3 449–458. 10.1111/j.1758-2229.2010.00223.x 23761307

[B18] Engelhardt M. A. Daly K. Swannell R. P. Head I. M. (2001). Isolation and characterization of a novel hydrocarbon-degrading, Gram-positive bacterium, isolated from intertidal beach sediment, and description of *Planococcus alkanoclasticus* sp. nov. *J. Appl. Microbiol.* 90 237–247. 10.1046/j.1365-2672.2001.01241 11168727

[B19] Faust K. Raes J. (2012). Microbial interactions: from networks to models. *Nat. Rev. Microbiol.* 10 538–549. 10.1038/nrmicro2832 22796884

[B20] Fox G. E. Wisotzkey J. D. Jurtshuk P. (1992). How close is close: 16S rRNA sequence identity may not be sufficient to guarantee species identity. *Int. J. Syst. Bacteriol.* 42 166–170. 10.1099/00207713-42-1-166 1371061

[B21] Friedman J. Alm E. J. (2012). Inferring correlation networks from genomic survey data. *PLoS Comput. Biol.* 8:e1002687 10.1371/journal.pcbi.1002687 PMC3447976 23028285

[B22] Glud R. N. Wenzhofer F. Middelboe M. Oguri K. Turnewitsch R. Canfield D. E. (2013). High rates of microbial carbon turnover in sediments in the deepest oceanic trench on Earth. *Nat. Geosci.* 6 284–288. 10.1038/NGEO1773

[B23] Guan H. X. Chen L. Y. Luo M. Liu L. H. Mao S. Y. Ge H. M. (2019). Composition and origin of lipid biomarkers in the surface sediments from the southern Challenger Deep, Mariana Trench. *Geosci. Front.* 10 351–360. 10.1016/j.gsf.2018.01.004

[B24] Gutierrez T. (2017). “Aerobic Hydrocarbon-Degrading Gammaproteobacteria: Xanthomonadales,” in *Taxonomy, Genomics and Ecophysiology of Hydrocarbon-Degrading Microbes*, ed. McGenity T. J. (Cham: Springer), 1–15. 10.1007/978-3-319-60053-6_4-1

[B25] Head I. M. Jones D. M. Röling W. F. (2006). Marine microorganisms make a meal of oil. *Nat. Rev. Microbiol.* 4 173–182. 10.1038/nrmicro1348 16489346

[B26] Hoffmann K. Hassenrück C. Salman-Carvalho V. Holtappels M. Bienhold C. (2017). Response of bacterial communities to different detritus compositions in arctic deep-sea sediments. *Front. Microbiol.* 8:266 10.3389/fmicb.2017.00266 PMC5323390 28286496

[B27] Hunt D. E. Lin Y. Church M. J. Karl D. M. Tringe S. G. Izzo L. K. (2013). Relationship between abundance and specific activity of bacterioplankton in open ocean surface waters. *Appl. Environ. Microbiol.* 79 177–184. 10.1128/AEM.02155-12 23087033 PMC3536108

[B28] Ichino M. C. Clark M. R. Drazen J. C. Jamieson A. Jones D. O. B. Martin A. P. (2015). The distribution of benthic biomass in hadal trenches: a modelling approach to investigate the effect of vertical and lateral organic matter transport to the seafloor. *Deep Sea Res. Part I Oceanogr. Res. Pap.* 100 21–33. 10.1016/j.dsr.2015.01.010

[B29] Jamieson A. J. (2011). “Ecology of deep oceans: hadal trenches,” in *Encyclopedia of Life Sciences (eLS)*, (Chichester: John Wiley and Sons, Ltd), 10.1002/9780470015902.a0023606

[B30] Jamieson A. J. (2015). *The Hadal Zone: Life in the Deepest Oceans.* Cambridge: Cambridge University Press.

[B31] Jamieson A. J. Fujii T. Mayor D. J. Solan M. Priede I. G. (2010). Hadal trenches: the ecology of the deepest places on Earth. *Trends. Ecol. Evol.* 25 190–197. 10.1016/j.tree.2009.09.009 19846236

[B32] Kearns P. J. Angell J. H. Howard E. M. Deegan L. A. Stanley R. H. Bowen J. L. (2016). Nutrient enrichment induces dormancy and decreases diversity of active bacteria in salt marsh sediments. *Nat. Commun.* 7:12881 10.1038/ncomms12881 PMC5052679 27666199

[B33] Kim M. Oh H. S. Park S. C. Chun J. (2014). Towards a taxonomic coherence between average nucleotide identity and 16S rRNA gene sequence similarity for species demarcation of prokaryotes. *Int. J. Syst. Evol. Microbiol.* 64 346–351. 10.1099/ijs.0.059774-0 24505072

[B34] La Cono V. Smedile F. Spada G. L. Arcadi E. Genovese M. Ruggeri G. (2015). Shifts in the meso- and bathypelagic archaea communities composition during recovery and short-term handling of decompressed deep-sea samples. *Environ. Microbiol. Rep.* 7 450–459. 10.1111/1758-2229.12272 25682761

[B35] La Cono V. Tamburini C. Genovese L. Spada G. L. Denaro R. Yakimov M. M. (2009). Cultivation-independent assessment of the bathypelagic archaeal diversity of Tyrrhenian Sea: comparative study of rDNA and rRNA-derived libraries and influence of sample decompression. *Deep Sea Res. Part II Top. Stud. Oceanogr.* 56 768–773. 10.1016/j.dsr2.2008.07.025

[B36] Landry Z. Swan B. K. Herndl G. J. Stepanauskas R. Giovannoni S. J. (2017). SAR202 genomes from the dark ocean predict pathways for the oxidation of recalcitrant dissolved organic matter. *mBio* 8:e00413-17 10.1128/mBio.00413-17 PMC5395668 28420738

[B37] León-Zayas R. Novotny M. Podell S. Shepard C. M. Berkenpas E. Nikolenko S. (2015). Single cells within the Puerto Rico trench suggest hadal adaptation of microbial lineages. *Appl. Environ. Microbiol.* 81 8265–8276. 10.1128/aem.01659-15 26386059 PMC4644660

[B38] Li R. Tun H. M. Jahan M. Zhang Z. Kumar A. Dilantha Fernando W. G. (2017). Comparison of DNA-, PMA-, and RNA-based 16S rRNA Illumina sequencing for detection of live bacteria in water. *Sci. Rep.* 7:5752 10.1038/s41598-017-02516-3 PMC5515937 28720878

[B39] Liang B. Wang L. Y. Mbadinga S. M. Liu J. F. Yang S. Z. Gu J. D. (2015). Anaerolineaceae and Methanosaeta turned to be the dominant microorganisms in alkanes-dependent methanogenic culture after long-term of incubation. *AMB Express* 5:37 10.1186/s13568-015-0117-4 PMC4469597 26080793

[B40] Liu J. Zheng Y. Lin H. Wang X. Li M. Liu Y. (2019). Proliferation of hydrocarbon-degrading microbes at the bottom of the Mariana Trench. *Microbiome* 7:47 10.1186/s40168-019-0652-3 PMC6460516 30975208

[B41] Liu R. Cheng K. H. Wong K. Cheng S. C. Lau S. C. (2015). Differential utility of the Bacteroidales DNA and RNA markers in the tiered approach for microbial source tracking in subtropical seawater. *Appl. Microbiol. Biotechnol.* 99 5669–5681. 10.1007/s00253-015-6410-y 25652655

[B42] Liu R. Wang L. Liu Q. F. Wang Z. X. Li Z. Z. Fang J. S. (2018a). Depth-resolved distribution of particle-attached and free-living bacterial communities in the water column of the new britain trench. *Front. Microbiol.* 9:625 10.3389/fmicb.2018.00625 PMC5893722 29670597

[B43] Liu R. Wang L. Wei Y. Fang J. (2018b). The hadal biosphere: recent insights and new directions. *Deep Sea Res. Part II Top. Stud. Oceanogr.* 155 11–18. 10.1016/j.dsr2.2017.04.015

[B44] Luo M. Glud R. N. Pan B. Wenzhöfer F. Xu Y. Lin G. (2018). Benthic carbon mineralization in hadal trenches: insights from in-situ determination of benthic oxygen consumption. *Geophys. Res. Lett.* 45 2752–2760. 10.1002/2017GL076232

[B45] Magoc T. Salzberg S. L. (2011). FLASH: fast length adjustment of short reads to improve genome assemblies. *Bioinformatics* 27 2957–2963. 10.1093/bioinformatics/btr507 21903629 PMC3198573

[B46] Maiväli Ü. Paier A. Tenson T. (2013). When stable RNA becomes unstable: the degradation of ribosomes in bacteria and beyond. *Biol. Chem.* 394 845–855. 10.1515/hsz-2013-0133 23612597

[B47] Matheus Carnevali P. B. Herbold C. W. Hand K. P. Priscu J. C. Murray A. E. (2018). Distinct microbial assemblage structure and archaeal diversity in sediments of arctic thermokarst lakes differing in methane sources. *Front. Microbiol.* 9:1192 10.3389/fmicb.2018.01192 PMC6000721 29930542

[B48] Mehrshad M. Rodriguez-Valera F. Amoozegar M. A. López-García P. Ghai R. (2018a). The enigmatic SAR202 cluster up close: shedding light on a globally distributed dark ocean lineage involved in sulfur cycling. *ISME J.* 12 655–668. 10.1038/s41396-017-0009-5 29208946 PMC5864207

[B49] Mehrshad M. Salcher M. M. Okazaki Y. Nakano S. I. Šimek K. Andrei A. S. (2018b). Hidden in plain sight-highly abundant and diverse planktonic freshwater Chloroflexi. *Microbiome* 6:176 10.1186/s40168-018-0563-8 PMC6169038 30285851

[B50] Meier-Kolthoff J. P. Auch A. F. Klenk H. P. Göker M. (2013). Genome sequence-based species delimitation with confidence intervals and improved distance functions. *BMC Bioinformatics* 14:60–60. 10.1186/1471-2105-14-60 23432962 PMC3665452

[B51] Mo Y. Zhang W. Yang J. Lin Y. Yu Z. Lin S. (2018). Biogeographic patterns of abundant and rare bacterioplankton in three subtropical bays resulting from selective and neutral processes. *ISME J.* 12 2198–2210. 10.1038/s41396-018-0153-6 29880912 PMC6092436

[B52] Morris J. H. Apeltsin L. Newman A. M. Baumbach J. Wittkop T. Su G. (2011). clusterMaker: a multi-algorithm clustering plugin for Cytoscape. *BMC Bioinformatics* 12:436 10.1186/1471-2105-12-436 PMC3262844 22070249

[B53] Mota M. J. Lopes R. P. Delgadillo I. Saraiva J. A. (2013). Microorganisms under high pressure-Adaptation, growth and biotechnological potential. *Biotechnol. Adv.* 31 1426–1434. 10.1016/j.biotechadv.2013.06.007 23831003

[B54] Nunoura T. Hirai M. Yoshida-Takashima Y. Nishizawa M. Kawagucci S. Yokokawa T. (2016). Distribution and niche separation of planktonic microbial communities in the water columns from the surface to the hadal waters of the Japan Trench under the Eutrophic Ocean. *Front. Microbiol.* 7:1261 10.3389/fmicb.2016.01261 PMC4978738 27559333

[B55] Nunoura T. Nishizawa M. Hirai M. Shimamura S. Harnvoravongchai P. Koide O. (2018). Microbial diversity in sediments from the bottom of the challenger deep, the Mariana Trench. *Microbes. Environ.* 33 186–194. 10.1264/jsme2.ME17194 29806625 PMC6031389

[B56] Nunoura T. Takaki Y. Hirai M. Shimamura S. Makabe A. Koide O. (2015). Hadal biosphere: insight into the microbial ecosystem in the deepest ocean on Earth. *Proc. Natl. Acad. Sci. U.S.A.* 112 E1230–E1236. 10.1073/pnas.1421816112 25713387 PMC4371994

[B57] Oren A. (2014). “The Family *Rhodocyclaceae*,” in *The Prokaryotes*, eds Rosenberg E. DeLong E. F. Lory S. Stackebrandt E. Thompson F. (Berlin: Springer), 975–998. 10.1007/978-3-642-30197-1_292

[B58] Peoples L. M. Donaldson S. Osuntokun O. Xia Q. Nelson A. Blanton J. (2018). Vertically distinct microbial communities in the Mariana and Kermadec trenches. *PLoS One* 13:e0195102 10.1371/journal.pone.0195102 PMC5886532 29621268

[B59] Peoples L. M. Grammatopoulou E. Pombrol M. Xu X. Osuntokun O. Blanton J. (2019). Microbial community diversity within sediments from two geographically separated Hadal Trenches. *Front. Microbiol.* 10:347 10.3389/fmicb.2019.00347 PMC6428765 30930856

[B60] Quast C. Pruesse E. Yilmaz P. Gerken J. Schweer T. Yarza P. (2013). The SILVA ribosomal RNA gene database project: improved data processing and web-based tools. *Nucleic Acids Res.* 41 D590–D596. 10.1093/nar/gks1219 23193283 PMC3531112

[B61] Reji L. Tolar B. B. Smith J. M. Chavez F. P. Francis C. A. (2019). Differential co-occurrence relationships shaping ecotype diversification within Thaumarchaeota populations in the coastal ocean water column. *ISME J.* 13 1144–1158. 10.1038/s41396-018-0311-x 30610232 PMC6474218

[B62] Roller B. R. Stoddard S. F. Schmidt T. M. (2016). Exploiting rRNA operon copy number to investigate bacterial reproductive strategies. *Nat. Microbiol.* 1:16160 10.1038/nmicrobiol.2016.160 PMC5061577 27617693

[B63] Salazar G. Cornejo-Castillo F. M. Benitez-Barrios V. Fraile-Nuez E. Álvarez-Salgado X. A. Duarte C. M. (2016). Global diversity and biogeography of deep-sea pelagic prokaryotes. *ISME J.* 10 596–608. 10.1038/ismej.2015.137 26251871 PMC4817678

[B64] Saw J. H. Nunoura T. Hirai M. Takaki Y. Parsons R. Michelsen M. (2019). Pangenomics reveal diversification of enzyme families and niche specialization in globally abundant SAR202 bacteria. *bioRxiv* [Preprint] 10.1101/692848 PMC6946804 31911493

[B65] Shannon P. Markiel A. Ozier O. Baliga N. S. Wang J. T. Ramage D. (2003). Cytoscape: a software environment for integrated models of biomolecular interaction networks. *Genome Res.* 13 2498–2504. 10.1101/gr.1239303 14597658 PMC403769

[B66] Smalley N. E. Taipale S. De Marco P. Doronina N. V. Kyrpides N. Shapiro N. (2015). Functional and genomic diversity of methylotrophic Rhodocyclaceae: description of *Methyloversatilis discipulorum* sp. nov. *Int. J. Syst. Evol. Microbiol.* 65 2227–2233. 10.1099/ijs.0.000190 26231539

[B67] Sogin M. L. Morrison H. G. Huber J. A. Mark Welch D. Huse S. M. Neal P. R. (2006). Microbial diversity in the deep-sea and the underexplored “rare biosphere”. *Proc. Natl. Acad. Sci. U.S.A.* 103 12115–12120. 10.1073/pnas.0605127103 16880384 PMC1524930

[B68] Stackebrandt E. (2006). “Defining taxonomic ranks,” in *The Prokaryotes*, eds Dworkin M. Falkow S. Rosenberg E. Schleifer K. H. Stackebrandt E. (New York, NY: Springer), 29–57. 10.1007/0-387-30741-9_3

[B69] Stackebrandt E. Ebers J. (2006). Taxonomic parameters revisited: tarnished gold standards. *Microbiol. Today* 33 152–155.

[B70] Steven B. Hesse C. Soghigian J. Gallegos-Graves V. Dunbar J. (2017). Simulated rRNA/DNA ratios show potential to misclassify active populations as dormant. *Appl. Environ. Microbiol.* 83:e00696-17 10.1128/AEM.00696-17 PMC5440720 28363969

[B71] Stewart H. A. Jamieson A. J. (2018). Habitat heterogeneity of hadal trenches: considerations and implications for future studies. *Prog. Oceanogr.* 161 47–65. 10.1016/j.pocean.2018.01.007

[B72] Stibal M. Schostag M. Cameron K. A. Hansen L. H. Chandler D. M. Wadham J. L. (2015). Different bulk and active bacterial communities in cryoconite from the margin and interior of the Greenland ice sheet. *Environ. Microbiol. Rep.* 7 293–300. 10.1111/1758-2229.12246 25405749

[B73] Sunagawa S. Coelho L. P. Chaffron S. Kultima J. R. Labadie K. Salazar G. (2015). Structure and function of the global ocean microbiome. *Science* 348:1261359 10.1126/science.1261359 25999513

[B74] Taira K. Yanagimoto D. Kitagawa S. (2005). Deep CTD casts in the Challenger Deep, Mariana Trench. *J. Oceanogr.* 61 447–454. 10.1007/s10872-005-0053-z

[B75] Tamburini C. Boutrif M. Garel M. Colwell R. R. Deming J. W. (2013). Prokaryotic responses to hydrostatic pressure in the ocean-a review. *Environ. Microbiol.* 15 1262–1274. 10.1111/1462-2920.12084 23419081

[B76] Tarn J. Peoples L. M. Hardy K. Cameron J. Bartlett D. H. (2016). Identification of free-living and particle-associated microbial communities present in hadal regions of the Mariana Trench. *Front. Microbiol.* 7:665 10.3389/fmicb.2016.00665 PMC4860528 27242695

[B77] Thrash J. C. Seitz K. W. Baker B. J. Temperton B. Gillies L. E. Rabalais N. N. (2017). Metabolic Roles of Uncultivated Bacterioplankton Lineages in the Northern Gulf of Mexico “Dead Zone”. *mBio* 8:e01017-17 10.1128/mBio.01017-17 PMC5596340 28900024

[B78] Wang Y. Gao Z. M. Li J. He L. S. Cui G. J. Li W. L. (2019). Hadal water sampling by in situ microbial filtration and fixation (ISMIFF) apparatus. *Deep Sea Res. Part I Oceanogr. Res. Pap.* 144 132–137. 10.1016/j.dsr.2019.01.009

[B79] Wenzhöfer F. Oguri K. Middelboe M. Turnewitsch R. Toyofuku T. Kitazato H. (2016). Benthic carbon mineralization in hadal trenches: assessment by in situ O2 microprofile measurements. *Deep Sea Res. Part I Oceanogr. Res. Pap.* 116 276–286. 10.1016/j.dsr.2016.08.013

[B80] Wheeler D. L. Barrett T. Benson D. A. Bryant S. H. Canese K. Chetvernin V. (2008). Database resources of the National Center for Biotechnology Information. *Nucleic Acids Res.* 36 D13–D21. 10.1093/nar/gkz899 18045790 PMC2238880

[B81] Williams R. J. Howe A. Hofmockel K. S. (2014). Demonstrating microbial co-occurrence pattern analyses within and between ecosystems. *Front. Microbiol.* 5:358 10.3389/fmicb.2014.00358 PMC4102878 25101065

[B82] Wolff T. (1959). The hadal community, an introduction. *Deep Sea Res.* 6 95–124. 10.1016/0146-6313(59)90063-2

[B83] Wolff T. (1970). The concept of the hadal or ultra-abyssal fauna. *Deep Sea Res. Oceanogr. Abstr.* 17 983–1003. 10.1016/0011-7471(70)90049-5

[B84] Yanagawa K. Morono Y. de Beer D. Haeckel M. Sunamura M. Futagami T. (2013). Metabolically active microbial communities in marine sediment under high-CO2 and low-pH extremes. *ISME J.* 7 555–567. 10.1038/ismej.2012.124 23096400 PMC3578575

[B85] Yilmaz P. Parfrey L. W. Yarza P. Gerken J. Pruesse E. Quast C. (2014). The SILVA and “All-species Living Tree Project (LTP)” taxonomic frameworks. *Nucleic Acids Res.* 42 D643–D648. 10.1093/nar/gkt1209 24293649 PMC3965112

[B86] Zeng Y. Feng F. Medová H. Dean J. Koblížek M. (2014). Functional type 2 photosynthetic reaction centers found in the rare bacterial phylum Gemmatimonadetes. *Proc. Natl. Acad. Sci. U.S.A.* 111 7795–7800. 10.1073/pnas.1400295111 24821787 PMC4040607

[B87] Zhang X. Xu W. Liu Y. Cai M. Luo Z. Li M. (2018). Metagenomics reveals microbial diversity and metabolic potentials of seawater and surface sediment from a Hadal Biosphere at the Yap Trench. *Front. Microbiol.* 9:2402 10.3389/fmicb.2018.02402 PMC6194347 30369913

