# Supplementary material for: Bulk and Active Sediment Prokaryotic Communities in the Mariana and Mussau Trenches
Source: Front Microbiol. 2020 Jul 14;11:1521. doi: 10.3389/fmicb.2020.01521 (PMC7381213; doi:10.3389/fmicb.2020.01521)
Supplement: Supplementary file 1 [file Data_Sheet_1.docx]

Supplementary Material

1. **Supplementary Methodology**

## Preparation of DNA and RNA NRCs

A 150-bp randomly generated nucleic acid sequence (GGGATTTCTC TGAGGATGTG CTTAGGAGCC CGTTTAAAAT GATGTTCACA CACGGAGTTG AGGATTTAAT AGCAGATGCG AGAATACTAC AGCTATAACT GGTCGGTGGA ACCACTGACT ATGGGCATAG CATAAGCAGG ATCATGTGGA) (Bartolini et al., 2007) was synthesized, inserted into Vector pBluescript II SK(+) (Takara, China) and transformed into *E.coli* Competent Cell JM109 (Takara, China). One positive clone was picked and plasmid was extracted. A 400 bp fragment containing synthesized sequence and necessary enzyme target sites was PCR amplified from the plasmid DNA. The amplicon was sequenced to confirm the sequences of the inserted DNA fragments, and then purified using MiniBEST Agarose Gel DNA Extraction Kit (Takara, China). The purified 400 bp PCR amplicon was used as DNA nucleic acid recovery control (DNA-NRC). To prepare for RNA-NRC, DNA-NRC was converted into a 383-bp recombinant RNA using in-vitro transcription (In vitro Transcription T7 Kit, Takara, China) and treated with DNase-I digesting and phenol-chloroform purification.

The copy numbers of DNA- and RNA-NRC were calculated using the formula: Copy no. per μl = (C × 6.022 × 10^23^) / (L ×10^9^ × 660 or 340), where C is the concentration of DNA-NRC or RNA-NRC (ng/μl), L is the length (number of bases) of DNA-NRC or RNA-NRC, 6.022×10^23^ is the Avogadro's number (molecules/mole), 660 is the average molecular weight (Da) of one pair of complimentary nucleotide bases in double-stranded DNA, and 340 is the average molecular weight of one nucleotide base in single-stranded RNA (Fey et al., 2004). DNA-NRC and RNA-NRC were diluted to 10^5^ copies/µl, divided into small aliquots, and stored under -80 °C until use.

## Quantitative PCR analysis

Each DNA and cDNA sample were tested with three SYBR® Green qPCR assays to determine copy numbers of bacterial and archaeal 16S rRNA and16S rDNA, as well as copy numbers of DNA and RNA NRCs (Supplementary Table S1-3). All qPCR assays were performed in 20 µl reactions, each contained 4 µl of DNA and 0.2 µM of each primer in 2X GoTaq® qPCR Master Mix (Promega). Duplicate reactions were prepared for each sample. The thermal cycles were performed on ABI Fast 7500 thermocycler (Applied Biosystems) using the following program: initial denaturation (95 ℃) for 5 min, followed by 40 cycles of denaturation (95 ℃) for 5 s, annealing at 60 ℃ for 30 s and extension at 72 ℃ for 40 s. Melting curves were included in each run to check the speciﬁcity of ampliﬁcation. Cycle of threshold (Ct) was calculated using auto-baseline and auto-threshold functions in ABI 7500 software. Standard curves of Ct vs. gene copy numbers were constructed using serial dilutions of plasmids containing amplicon fragment of each gene marker. The plasmids were produced by cloning of the PCR amplicons of the corresponding assays using the TOPO TA cloning kit (Invitrogen). The presence of PCR inhibitors in DNA and cDNA samples was determined based on the difference in the Ct values between two successive 10-fold dilutions of a sample (ΔCt). Inhibition was considered significant when ΔCt was <3 (Dick et al. 2010). If obvious inhibitions occurred, the sample were further diluted. In addition, to estimate the combined effects of nucleic acid extraction inefficiency and PCR inhibition, the percent recovery of the NRC was determined for each sample by dividing the measured copy numbers with total copies added (1× 10^6^ copies). The copy numbers of the bacterial and archaea 16S rRNA gene markers reported in the manuscript have been corrected by the values of percent recovery obtained for each sample.

## Filtered contaminant taxa from the OTU tables

Sequences found within our sequenced PCR negative control and similar to known contaminants related with human sources or contaminants reported in previous hadal trench studies (Nunoura et al., 2015; Peoples et al., 2019) were removed from the generated OTU table. The removed taxa including *Bifidobacterium, Enhydrobacter, Eubacterium*; *Acinetobacter, Stenotrophomonas, Methylobacterium, Rhizobium, Escherichia-Shigella, Massilia, Ralstonia, Deinococcus, Anoxybacillus, Brevundimonas, Enterobacter, Streptococcus, Burkholderia-Paraburkholderia, Staphylococcus, Sphingomonas, Sphingobium, Sphingobacterium, Bradyrhizobium, Paenibacillus, Akkermansia, Bacteroides,Aeromonas, Neisseria, Leptotrichia, Dermabacter, Alkanindiges, Corynebacterium_1, Campylobacter, Bifidobacterium, Peptoclostridium,* and families Comamonadaceae, Erysipelotrichaceae, Lachnospiraceae, Ruminococcaceae, Bacteroidales_S24-7_group, Prevotellaceae, Peptoniphilus, Cloacibacterium, Epilithonimonas and Neisseriaceae.

1. **Supplementary Figures and Tables**

## Supplementary Figures


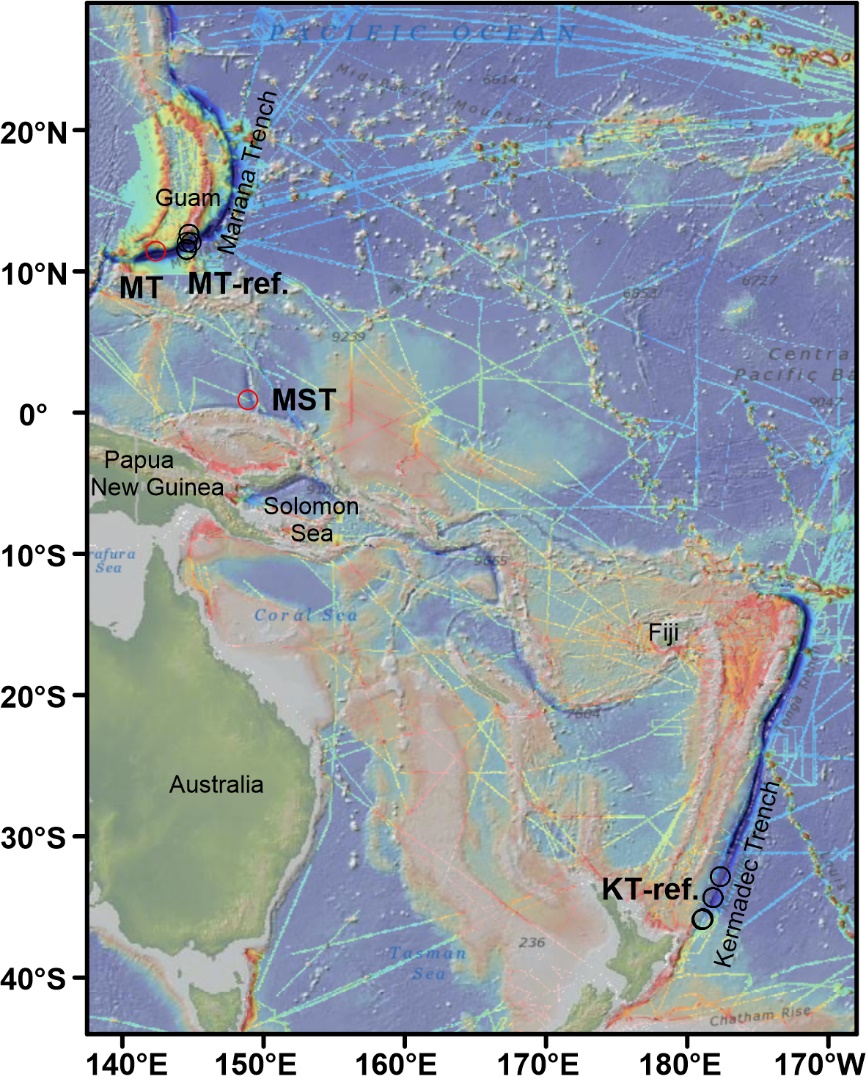


**Supplementary Figure S1.** Map of sampling sites in this study and those in Peoples et al. (2019). The red circles show sites of Mariana Trench (MT) and Mussau Trench (MST) sampled in this study. Black circles show sampling sites of Mariana (MT-ref.) and Kermadec trenches (KT-ref.) in Peoples et al. (2019). Base map was adopted from the National Oceanic and Atmospheric Administration (NOAA) (accessed via <https://maps.ngdc.noaa.gov> /viewers/bathymetry/).

**Supplementary Figure S2.** Variations of major alpha diversity indexes between different depths (0-10 cm) and between different molecular markers (DNA and cDNA) in the sediment of the two hadal trenches.


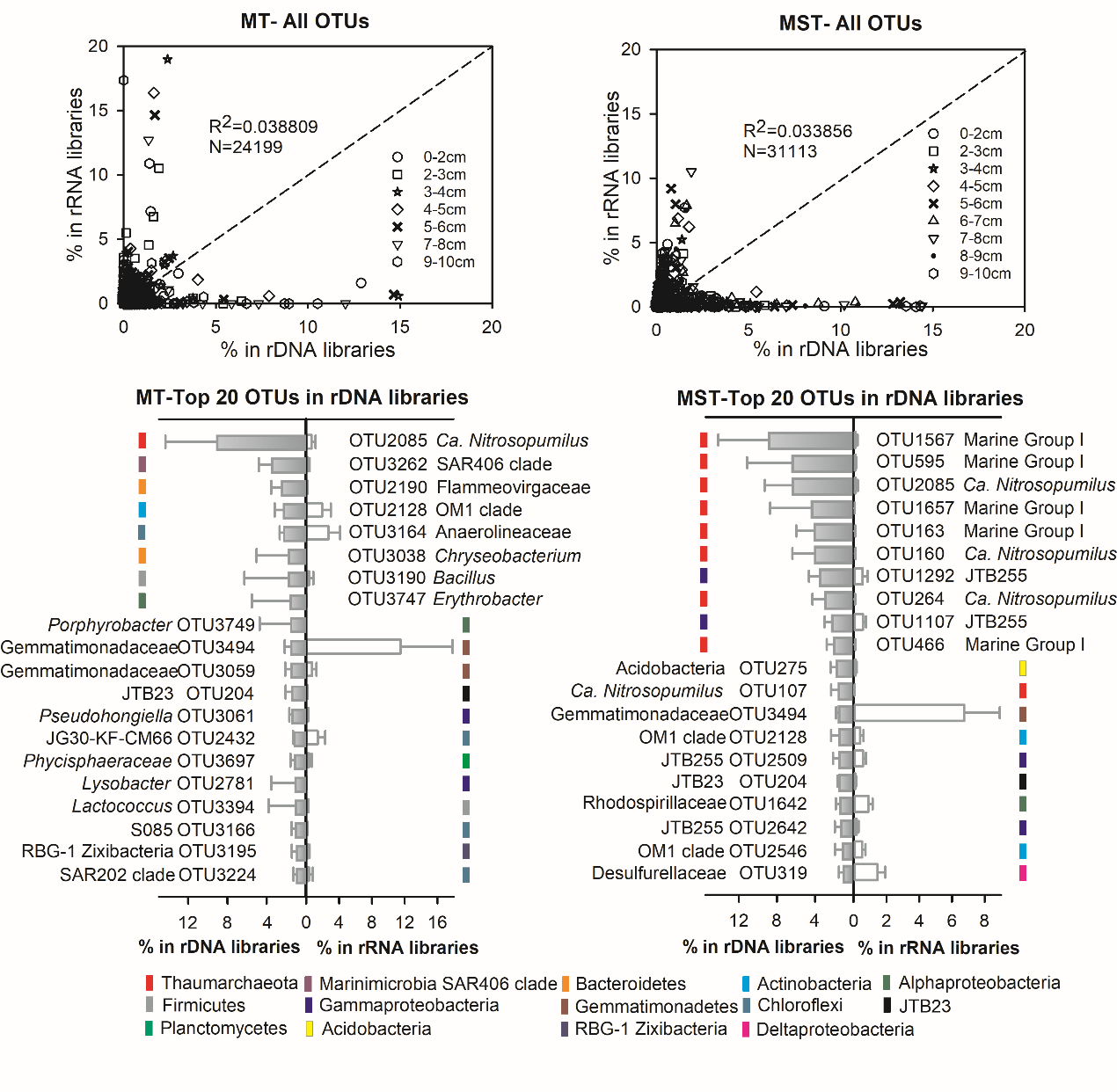


**Supplementary Figure S3.** The top 20 most dominant OTUs in rDNA libraries and their relative abundance in all rDNA or rRNA libraries of sediment microbial communities of the Mariana and Mussau trenches. Grey and white bars show the average relative abundance of each OTU in rDNA and rRNA libraries of the same trench, respectively. Error bars show standard deviation of the relative abundance. OTU name and the lowest recognized classification were labeled, and the colored bars indicate the phylum/class of the OTUs.


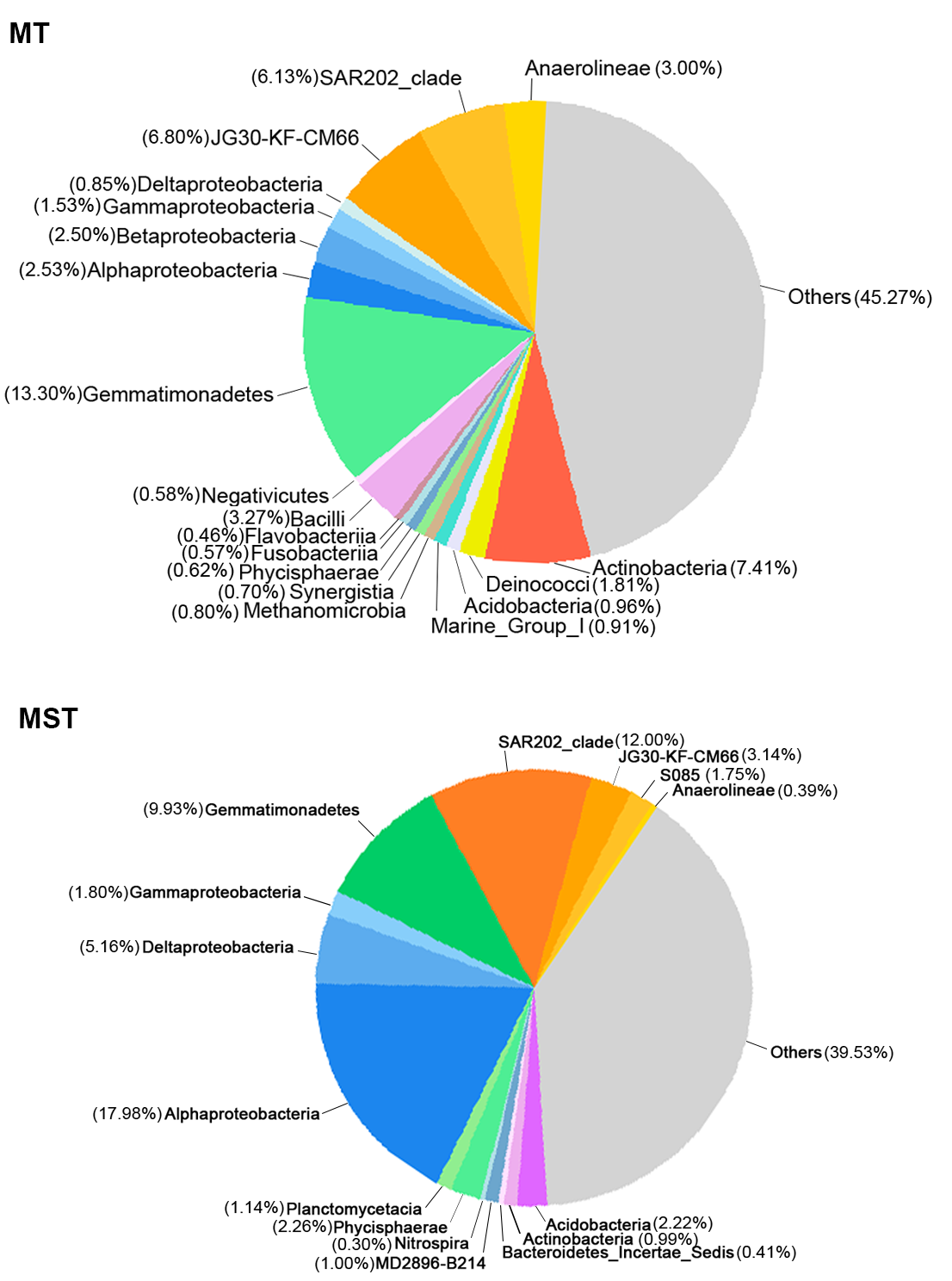


**Supplementary Figure S4.** Taxonomy of the dominant OTUs in rRNA libraries of MT sediments at class level. Dominant OTUs are defined as those averagely account for ≥ 0.3% of total sequences among all rRNA libraries from the same trench. Numbers in brackets show the average relative abundance of the dominant OTUs from particular classes. OTUs that were not identified as dominant were grouped into “Others”.


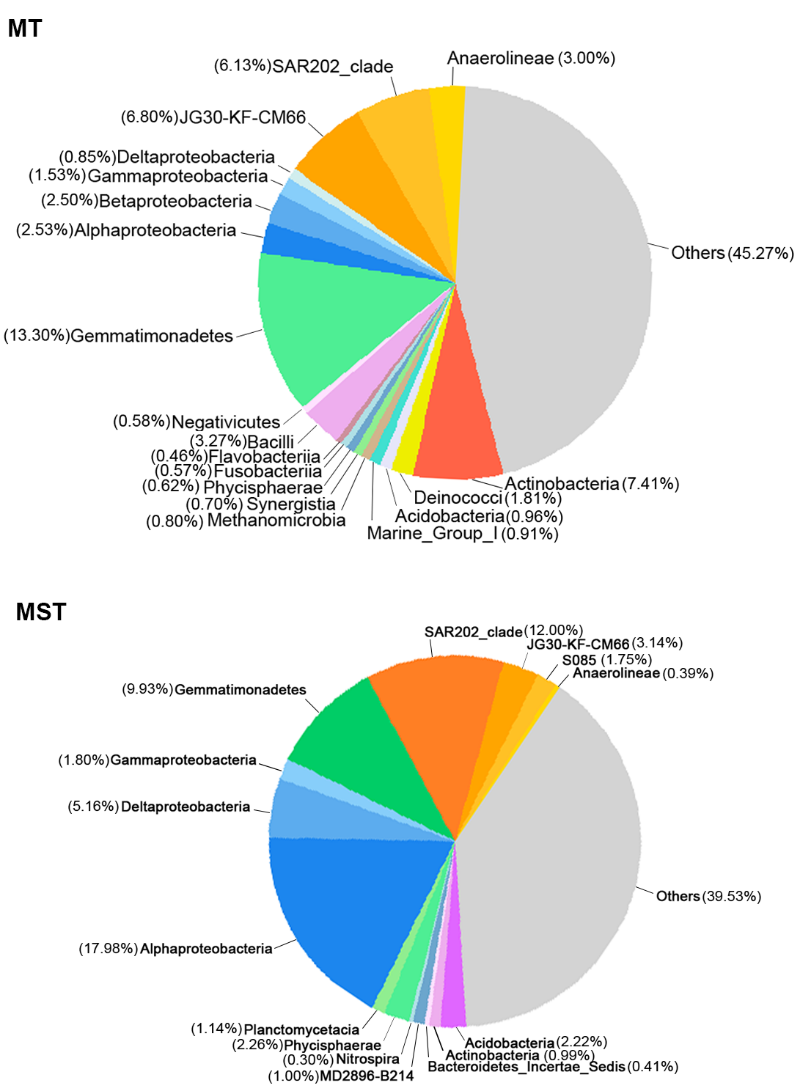


**Supplementary Figure S5.** Taxonomy of the dominant OTUs in rRNA libraries of MST sediments at class level. Dominant OTUs are defined as those averagely account for ≥ 0.3% of total sequences among all rRNA libraries from the same trench. Numbers in brackets show the average relative abundance of the dominant OTUs from particular classes. OTUs that were not identified as dominant were grouped into “Others”.

## Supplementary Tables

**Supplementary Table S1.** Primers utilized in this study.

| Primers | Targets | Purpose | Sequence (5’ - 3’) | Annealing temperature (°C) | Amplicon size (bp) | References |
| --- | --- | --- | --- | --- | --- | --- |
| 515F | Bacteria and archaea | Sequencing | GTGCCAGCMGCCGCGG | 55 | 392 | Caporaso et al., [2011](https://www.ncbi.nlm.nih.gov/pmc/articles/PMC5797777/#B9)  Armitage et al., [2012](https://www.ncbi.nlm.nih.gov/pmc/articles/PMC5797777/#B3) |
| 907R |  |  | CCGTCAATTCMTTTRAGTTT |  |  |  |
|  |  |  |  |  |  |  |
| Eub338F | Bacteria | qPCR | ACTCCTACGGGAGGCAGCAG | 60 | 174 | Thijs et al., 2017 |
| Eub518R |  |  | ATTACCGCGGCTGCTGG |  |  |  |
|  |  |  |  |  |  |  |
| Uni519F | Archaea | qPCR | CAGCMGCCGCGGTAA | 60 | 389 | Jørgensen et al., 2012 |
| Arch908R |  |  | CCCGCCAATTCCTTTAAGTT |  |  |  |
|  |  |  |  |  |  |  |
| NRC-F | Nucleic acid recovery control | qPCR | GGGATTTCTCTGAGGATGTGCTTA | 60 | 48 | Bartolini et al., 2007 |
| NRC-R |  |  | TCCACATGATCCTGCTTATGCTAT |  |  |  |

**Supplementary Table S2.** In-silico tests on specificity and coverage of all primer pairs, using the “Test Probe” tool in the Silva rRNA database.

| Primer | Silva database (SSU-132 REF) | | |
| --- | --- | --- | --- |
|  | Allowing 0 mismatch | Allowing 1 mismatch | Allowing 2 mismatch |
| 515F/907R | 0.5%Archaea | 86.8%Archaea | 93.4%Archaea |
|  | 86.9%Bacteria | 95.7%Bacteria | 96.8%Bacteria |
|  | 70.4%Eukaryota | 92.6%Eukaryota | 94.8%Eukaryota |
| Eub338F/Eub518R | 0.0%Archaea | 0.0%Archaea | 0.1%Archaea |
|  | 81.6%Bacteria | 93.0%Bacteria | 95.9%Bacteria |
|  | 0.0%Eukaryota | 0.1%Eukaryota | 0.6%Eukaryota |
| Uni519F/Arch908R | 84.7%Archaea | 92.8%Archaea | 94.1%Archaea |
|  | 0.0%Bacteria | 0.8%Bacteria | 73.7%Bacteria |
|  | 0.0%Eukaryota | 0.8%Eukaryota | 74.9%Eukaryota |

**Supplementary Table S3.** Performance characteristics of qPCR assays. Four standard curves were obtained for each assay during the course of this study. The values of R^2^, slope, efficiency was determined for each curve. Data shown for each assay are the range and mean (in parenthesis) of the values of the 4 curves. The mean coefficient of variation (CV) for each assay was calculated using Ct values from all 4 curves. The 4 curves of each assay had the same dynamic range.

|  | Assay | | |
| --- | --- | --- | --- |
|  | NRC | Bac | Arch |
| No. of standard curves | 4 | 4 | 4 |
| R^2^ | 0.985 - 0.996 | 0.986 - 0.994 | 0.981 - 0.998 |
|  | (0.992) | (0.991) | (0.990) |
| Efficiency (%) | 92.2 - 97.0 | 108.0 - 128.6 | 89.7 - 95.5 |
|  | (95.7) | (117.1) | (92.2) |
| Mean CV (%) | 8.39 | 2.46 | 6.33 |

**Supplementary Table S4.** Alpha diversity indexes of the rDNA and rRNA libraries from sediments of the two trenches.

| Samples | Depth (cmbsf) | Sobs | Shannon | Simpson | Ace | Chao | Coverage |
| --- | --- | --- | --- | --- | --- | --- | --- |
| MT_DNA | 0-2 | 701 | 5.1745 | 0.0232 | 915 | 918 | 0.9663 |
|  | 2-3 | 363 | 4.9105 | 0.0151 | 401 | 406 | 0.9910 |
|  | 3-4 | 680 | 4.9903 | 0.0301 | 916 | 917 | 0.9654 |
|  | 4-5 | 399 | 5.0397 | 0.0150 | 441 | 441 | 0.9906 |
|  | 5-6 | 633 | 4.9844 | 0.0301 | 761 | 741 | 0.9756 |
|  | 6-7 | 591 | 4.9856 | 0.0248 | 706 | 723 | 0.9772 |
|  | 7-8 | 604 | 4.7957 | 0.0300 | 772 | 783 | 0.9720 |
|  | 8-9 | 486 | 5.0440 | 0.0189 | 538 | 541 | 0.9873 |
|  | 9-10 | 216 | 4.0232 | 0.0397 | 242 | 248 | 0.9945 |
| MT_cDNA | 0-2 | 251 | 4.7866 | 0.0150 | 260 | 264 | 0.9972 |
|  | 2-3 | 225 | 4.4426 | 0.0279 | 231 | 232 | 0.9979 |
|  | 3-4 | 369 | 4.4940 | 0.0452 | 393 | 394 | 0.9927 |
|  | 4-5 | 387 | 4.7663 | 0.0349 | 398 | 401 | 0.9952 |
|  | 5-6 | 348 | 4.6810 | 0.0309 | 369 | 379 | 0.9936 |
|  | 7-8 | 365 | 5.0794 | 0.0213 | 380 | 398 | 0.9948 |
|  | 9-10 | 148 | 3.9458 | 0.0512 | 150 | 150 | 0.9991 |
| MST_DNA | 0-2 | 685 | 4.8112 | 0.0232 | 1447 | 1228 | 0.9521 |
|  | 2-3 | 736 | 5.0896 | 0.0159 | 1500 | 1190 | 0.9509 |
|  | 3-4 | 680 | 4.8701 | 0.0192 | 1619 | 1189 | 0.9512 |
|  | 4-5 | 598 | 4.7067 | 0.0222 | 1408 | 1062 | 0.9576 |
|  | 5-6 | 440 | 3.8930 | 0.0538 | 1066 | 798 | 0.9681 |
|  | 6-7 | 558 | 4.4211 | 0.0342 | 1171 | 899 | 0.9624 |
|  | 7-8 | 527 | 4.3063 | 0.0451 | 1044 | 825 | 0.9660 |
|  | 8-9 | 559 | 4.3311 | 0.0441 | 1189 | 892 | 0.9623 |
|  | 9-10 | 485 | 4.0039 | 0.0640 | 1027 | 776 | 0.9678 |
| MST_cDNA | 0-2 | 774 | 5.4232 | 0.0116 | 1072 | 1066 | 0.9590 |
|  | 2-3 | 840 | 5.4861 | 0.0108 | 1223 | 1188 | 0.9524 |
|  | 3-4 | 826 | 5.4883 | 0.0107 | 1586 | 1293 | 0.9484 |
|  | 4-5 | 674 | 5.1466 | 0.0163 | 1117 | 917 | 0.9635 |
|  | 5-6 | 622 | 4.9771 | 0.0221 | 1049 | 913 | 0.9653 |
|  | 6-7 | 688 | 5.1162 | 0.0178 | 1282 | 1039 | 0.9585 |
|  | 7-8 | 661 | 5.0466 | 0.0206 | 928 | 920 | 0.9642 |
|  | 8-9 | 754 | 5.2315 | 0.0146 | 1464 | 1148 | 0.9525 |
|  | 9-10 | 747 | 5.3004 | 0.0141 | 1080 | 1118 | 0.9572 |

**Supplementary Table S5.** Identified keystone OTUs from co-occurrence network.

| OTU | Phylum | Class | Order | Family | Genus |
| --- | --- | --- | --- | --- | --- |
| *Top 10 OTUs with highest Betweenness centrality scores* | | | | | |
| OTU1792 | Chloroflexi | SAR202 clade | Norank SAR202 | Norank | Norank |
| OTU3180 | Chloroflexi | SAR202 clade | Norank SAR202 | Norank | Norank |
| OTU185 | Proteobacteria | Gammaproteobacteria | BD7-8 marine group | Norank | Norank |
| OTU3165 | Chloroflexi | JG30-KF-CM66 | Norank | Norank | Norank |
| OTU1973 | Planctomycetes | Phycisphaerae | Phycisphaerales | Phycisphaeraceae | Urania-1B-19 marine sediment group |
| OTU1434 | Proteobacteria | Alphaproteobacteria | Rhodobacterales | Rhodobacteraceae | Norank |
| OTU3146 | Chloroflexi | SAR202 clade | Norank | Norank | Norank |
| OTU1415 | Proteobacteria | Alphaproteobacteria | Rhodospirillales | Rhodospirillaceae | Norank |
| OTU196 | Chloroflexi | SAR202 clade | Norank | Norank | Norank |
| OTU2509 | Proteobacteria | Gammaproteobacteria | Xanthomonadales | JTB255 marine benthic group | Norank |
| *Top 10 OTUs with highest Degree centrality scores* | | | | | |
| OTU2128 | Actinobacteria | Actinobacteria | Acidimicrobiales | OM1 clade | Norank |
| OTU1415 | Proteobacteria | Alphaproteobacteria | Rhodospirillales | Rhodospirillaceae | Norank |
| OTU3164 | Chloroflexi | Anaerolineae | Anaerolineales | Anaerolineaceae | Norank |
| OTU3165 | Chloroflexi | JG30-KF-CM66 | Norank | Norank | Norank |
| OTU1363 | Proteobacteria | Alphaproteobacteria | Rhodospirillales | Rhodospirillaceae | Norank |
| OTU368 | Proteobacteria | Alphaproteobacteria | Rhodospirillales | Rhodospirillaceae | Norank |
| OTU3140 | Chloroflexi | JG30-KF-CM66 | Norank | Norank | Norank |
| OTU2848 | Chloroflexi | SAR202 clade | Norank | Norank | Norank |
| OTU1107 | Proteobacteria | Gammaproteobacteria | Xanthomonadales | JTB255 marine benthic group | Norank |
| OTU2509 | Proteobacteria | Gammaproteobacteria | Xanthomonadales | JTB255 marine benthic group | Norank |

1. **References**

Armitage, D., Gallagher, K., Youngblut, N., Buckley, D., & Zinder, S. (2012). Millimeter-scale patterns of phylogenetic and trait diversity in a salt marsh microbial mat. Front. Microbiol. 3:293. doi: 10.3389/fmicb.2012.00293

Bartolini, B., Garbuglia, A. R., Horejsh, D., Martini, F., Carletti, F., & Capobianchi, M. R. (2007). Simultaneous control of DNA and RNA processing efficiency using a nucleic acid calibration set. Bio Techniques 42, 452-456. doi: 10.2144/000112450

Caporaso, J. G., Lauber, C. L., Walters, W. A., Berg-Lyons, D., Lozupone, C. A., Turnbaugh, P. J., et al. (2011). Global patterns of 16S rRNA diversity at a depth of millions of sequences per sample. Proc. Natl. Acad. Sci. U.S.A. 108, 4516-4522. doi: 10.1073/pnas.1000080107

Dick, L.K., Stelzer, E., Bertke, E., Fong, D.L., Stoeckel, D.M. (2010) Relative decay of Bacteroidales microbial source tracking markers and cul- tivated Escherichia coli in freshwater microcosms. Appl. Environ. Microbiol. 76:3255–3262

Fey, A., Eichler, S., Flavier, S., Christen, R., Höfle, M. G., & Guzmán, C. A. (2004). Establishment of a real-time PCR-based approach for accurate quantification of bacterial RNA targets in water, using *Salmonella* as a model organism. Appl. Environ. Microbiol. 70, 3618-3623. doi: 10.1128/AEM.70.6.3618-3623.2004

Nunoura, T., Takaki, Y., Hirai, M., Shimamura, S., Makabe, A., Koide, O., et al. (2015). Hadal biosphere: insight into the microbial ecosystem in the deepest ocean on Earth. Proc. Natl. Acad. Sci. U.S.A. 112, E1230–E1236. doi: 10.1073/pnas.1421816112

Jørgensen, S. L., Hannisdal, B., Lanzén, A., Baumberger, T., Flesland, K., Fonseca, R., et al. (2012). Correlating microbial community profiles with geochemical data in highly stratified sediments from the Arctic Mid-Ocean Ridge. Proc. Natl. Acad. Sci. U.S.A. 109, E2846–E2855. doi: 10.1073/pnas.1207574109

Peoples, L. M., Grammatopoulou, E., Pombrol, M., Xu, X., Osuntokun, O., Blanton, J., et al. (2019). Microbial Community Diversity Within Sediments From Two Geographically Separated Hadal Trenches. Front. Microbiol. 10:347. doi: 10.3389/fmicb.2019.00347

Thijs, S., Op De Beeck, M., Beckers, B., Truyens, S., Stevens, V., Van Hamme, J. D., et al. (2017). Comparative Evaluation of Four Bacteria-Specific Primer Pairs for 16S rRNA Gene Surveys. Front. Microbiol. 8:494. doi: 10.3389/fmicb.2017.00494
